# Targeting CIRP and IL-6R-mediated microglial inflammation to improve outcomes in intracerebral hemorrhage

**DOI:** 10.1016/j.jare.2025.09.012

**Published:** 2025-09-09

**Authors:** Lisha Ye, Xiaoyan Tang, Fangming Liu, Tianjiao Wei, Ting Xu, Zhenglin Jiang, Lihua Xu, Chao Xiang, Xiaoyu Yuan, Lihua Shen, Jianjun Gu, Qianqian Luo, Guohua Wang

**Affiliations:** a Department of Neurophysiology and Neuropharmacology, Institute of Special Environmental Medicine and Co-innovation Center of Neuroregeneration, Nantong University, 9 Seyuan Road, Nantong, Jiangsu 226019, China; b Beijing Institute of Brain Disorders, No. 10 Xitoutiao, Beijing 100069, China; c Department of Neurosurgery, Zhengzhou University People’s Hospital (Henan Provincial People’s Hospital), 7 Weiwu Road, Zhengzhou, Henan 450000, China; d Department of Emergency, Affiliated Hospital of Nantong University, Nantong 226001, China; e Department of Neurology, Affiliated Hospital of Nantong University, Nantong 226001, China; f Department of Neurology, Xuanwu Hospital, Capital Medical University, 45 Changchun Street, Beijing 100053, China

**Keywords:** Intracerebral hemorrhage, Cold-inducible RNA-binding proteins, Neuroinflammation, Microglial polarization, Tat peptides

## Abstract

**Introduction:**

Cold-inducible RNA-binding protein (CIRP) is an emerging inflammatory mediator implicated in neuronal injury following intracerebral hemorrhage (ICH). However, the underlying mechanisms by which CIRP contributes to neuroinflammation remain unclear.

**Objectives:**

To investigate the role of neuron-derived CIRP in activating microglial inflammation via IL-6 receptor (IL-6R) signaling after ICH and to evaluate the therapeutic potential of targeting CIRP and IL-6R using a designed peptide inhibitor.

**Methods:**

Single-cell RNA sequencing was performed to identify key genes and pathways involved in microglial responses post-ICH. A peptide inhibitor, Tat-CIRP-CMA (TCC), was designed to target CIRP and IL-6R and tested in both *in vitro* (oxygen-glucose deprivation model) and *in vivo* (rat ICH model) systems. The effects of TCC on neuronal CIRP release, microglial activation, inflammation, and phagocytic function were assessed. A microglia-specific IL-6Rα knockout mouse model was used to further validate the functional relevance of the IL-6R/ signal transducer and activator of transcription 3 (STAT3) pathway. Clinical analysis of human ICH patients evaluated the correlation between peripheral CIRP expression and infarct volume.

**Results:**

Neuron-derived CIRP activates microglia through IL-6R and downstream STAT3 signaling, driving pro-inflammatory responses in ICH. TCC treatment significantly reduced neuronal CIRP release, suppressed microglial activation, and decreased inflammatory cytokine levels. TCC inhibited the IL-6R /STAT3 pathway and enhanced microglial phagocytosis of red blood cells. Microglia-specific IL-6Rα deletion mirrored the anti-inflammatory and neuroprotective effects observed with TCC, reducing hematoma volume and improving sensory and behavioral outcomes. In human ICH patients, elevated peripheral CIRP expression positively correlated with infarct volume, supporting its value as a biomarker for ICH severity and prognosis.

**Conclusion:**

CIRP plays a pivotal role in post-ICH neuroinflammation by acting on IL-6R in microglia. The TCC peptide inhibitor effectively reduces CIRP-IL-6R mediated inflammation and supports neuroprotection. CIRP represents a promising therapeutic target and biomarker for ICH.

## Highlights

• Single-cell RNA-seq identified key inflammatory genes in microglia after ICH.
• CIRP promotes neuroinflammation after ICH via microglial IL-6R/STAT3 signaling.
• Targeting CIRP-IL-6R interaction reduced CIRP release and microglial activation.
• IL-6Rα deletion in microglia replicated anti-inflammatory and protective effects.
• CIRP levels in blood were positively correlated with infarct volume in ICH patients.

## Graphical abstract

This study illustrates that targeting Cold-inducible RNA-binding protein (CIRP) and IL-6 receptor can significantly attenuate neuroinflammation and enhance recovery following ICH. ① Intracerebral hemorrhage (ICH) occurs, ② leading to neuronal injury. ③ The damaged neurons release cold-inducible RNA-binding protein (CIRP), ④ which stimulates the activation of microglia. ⑤ CIRP can also bind to interleukin-6 receptor (IL-6R) on the microglial cell membrane, ⑥–⑦ resulting in the phosphorylation of STAT3 within the microglial nucleus. ⑧ This interaction promotes the polarization of microglia toward a pro-inflammatory phenotype, ⑨ thereby amplifying the microglial inflammatory response. ⑩ The excessive inflammation, in turn, exacerbates neuronal damage, forming a vicious cycle.

It demonstrates that the CIRP-targeting peptide Tat-CIRP CMA (TCC) effectively reduces inflammatory responses by inhibiting the IL-6R/JAK/STAT3 pathway, modulating microglial polarization, and enhancing neuronal protection.

**Figure:**
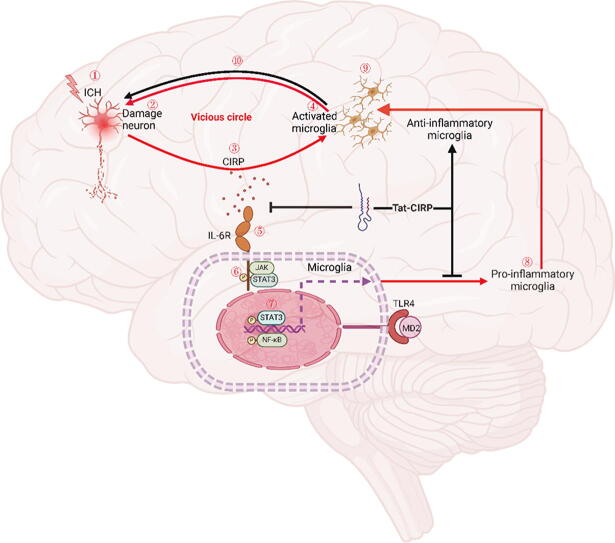

## Introduction

Intracerebral hemorrhage (ICH), a subtype of hemorrhagic stroke, is strongly associated with hypertension, identified as the leading risk factor in 50 % of cases[1,2]. Both the incidence and mortality rates of ICH have been rising annually[3], and the outcomes are often devastating—only 20 % of survivors regain the ability to perform self-care within six months, placing significant burdens on patients and their families[1,4]. Primary brain injury in ICH is caused by hematoma expansion and increased intracranial pressure, while secondary injury, driven by inflammatory processes, plays a critical role in the deterioration of neurological function[5,6]. As the brain is normally a sterile organ, the presence of blood components in the parenchyma can induce cytotoxicity, excitotoxicity, oxidative stress, and inflammatory responses[7,8].

Microglia, the primary immune cells in the brain, make up 10–15 % of all neuroglial cells[9]. These cells are among the first responders to pathological stimuli such as infection, brain injury, and neurodegeneration, including after ICH[[10], [11], [12]]. Activated microglia release large amounts of pro-inflammatory cytokines, such as interleukin-1 beta (IL-1β), interleukin-6 (IL-6), tumor necrosis factor-alpha (TNF-α), as well as nitric oxide and reactive oxygen species (ROS), which contribute to neuronal damage[[13], [14], [15], [16], [17], [18]]. Microglia can polarize into either a pro-inflammatory M1 state or an anti-inflammatory M2 state, with M1 promoting tissue damage and M2 facilitating repair[19,20]. Our previous studies have shown that post-injury inflammatory responses are predominantly mediated by microglia, which can switch from an initial M2 state to a more sustained M1 state[20,21]. This dynamic polarization complicates the efficacy of conventional steroidal and non-steroidal anti-inflammatory drugs, which broadly suppress microglial activation and impair their ability to clear debris[22,23]. Early inhibition of microglial activation may improve outcomes after ICH, but prolonged suppression could diminish the beneficial effects of microglia. Thus, modulating microglial polarization is essential for managing neuroinflammatory responses and mitigating secondary brain injury[16,23].

Cold-inducible RNA-binding protein (CIRP or called CIRBP), a 172-amino acid stress protein, plays a role in regulating various cellular processes, including growth, senescence, and apoptosis, potentially through post-transcriptional regulation[24,25]. Extracellular CIRP (eCIRP), recognized as a damage-associated molecular pattern (DAMP), is released into circulation during conditions such as hemorrhagic shock and sepsis, where it binds to myeloid differentiation protein 2 (MD2) and Toll-like receptor 4 (TLR4), activating downstream NF-κB signaling and promoting the release of pro-inflammatory cytokines[26,27]. Under normal conditions, CIRP expression in the brain is low; however, it becomes significantly upregulated in conditions such as ischemic stroke[28], and induces cytotoxicity and inflammation in a TLR4-dependent manner during sepsis[29]. These findings suggest that CIRP released from damaged neurons acts as a DAMP, driving neuroinflammation and exacerbating injury following hemorrhage.

Interleukin-6 receptor (IL-6R) is a transmembrane receptor that mediates crucial inflammatory and immune signaling cascades. Upon binding to its ligand IL-6, IL-6R forms a complex with glycoprotein 130 (gp130), activating downstream signaling pathways, which regulates the transcription of pro-inflammatory genes[30]. Emerging evidence has linked elevated IL-6 and IL-6R signaling to neuroinflammation, increased blood–brain barrier permeability, and exacerbated secondary brain injury following ICH[[30], [31], [32]]. Clinical and experimental studies have reported elevated IL-6 levels correlating with larger hematoma volumes, neurological deterioration, and poor clinical outcomes in ICH patients. Additionally, animal studies indicate that inhibition or genetic deletion of IL-6R can attenuate inflammatory responses, decrease neuronal apoptosis, and promote hematoma resolution, thereby improving neurological recovery[33]. Emerging evidence suggests that CIRP can also directly interact with IL-6Rα, potentially contributing to alternative inflammatory cascades, such as the IL-6R/STAT3 axis [34], suggesting additional downstream inflammatory pathways beyond TLR4. Despite these findings, the precise cellular and molecular interactions involving IL-6R in ICH-induced neuroinflammation, particularly its potential interplay with neuron-derived factors such as CIRP, remain poorly defined, warranting further investigation.

Qiang X et al., in a *Nature Medicine* study demonstrated that neutralizing CIRP with anti-CIRP antibodies significantly reduced systemic and local inflammation in hemorrhagic shock and sepsis[26]. Furthermore, CIRP inhibition has shown robust neuroprotective effects in rodent and nonhuman primate stroke models[25], while our prior research demonstrated the therapeutic potential of the Tat-C16 peptide in mitigating hypoxia-induced cognitive decline[35].

Building on this foundation, in the present study, we designed novel synthetic peptides to counteract excessive CIRP release, competitively antagonize the amino acid sequence mediating the binding between CIRP and IL-6R, modulate microglial activation, and attenuate central nervous system inflammation. By elucidating CIRP’s role in neuroinflammation and exploring its downstream signaling pathways, we aim to identify new therapeutic strategies to inhibit secondary injury in ICH and improve patient outcomes.

## Materials and methods

### Experimental animals

All animal experiments were complied with the ARRIVE guidelines. Male Sprague-Dawley (SD) rats, aged 8 weeks and weighing 220–230 g, were provided by the Laboratory Animal Center of Nantong University (SYXK(SU)-2012-0030). The animals were housed, managed, and used in strict accordance with the Nantong University Laboratory Animal Management Guidelines for the Care and Use of Experimental Animals of the National Institutes of Health and approved by the Animal Ethics Committee of Nantong University (S20201115-902). C57BL/6 male mice (8–12 weeks old), weighing 22–28 g, were provided by the Experimental Animal Center of Nantong University. *Cirbp*^−/−^ (S-KO-01510) and *IL-6Rα*^fl/fl^ (S-CKO-17651) mice were purchased from Cyagen Biosciences Inc[35]. *Cx3cr1*^CreERT2^ mice (Stock number: 021160) supplied by Jackson Laboratory (Bar Harbor, ME, USA) were kindly provided by Professor Yong-Jing Gao at Nantong University[36]. *Cx3cr1*^CreERT2/+^ mice and *IL-6Rα*^fl/fl^ mice were bred in-house to generate *Cx3cr1*^CreERT2/+^:: *IL-6Rα*^fl/fl^ mice, the offsprings of which termed as *IL-6Rα^MG-KO^* after intraperitoneal injections of tamoxifen (T5648, Sigma-Aldrich, MO, USA; 75 mg/kg daily for 5 days) treatment. Age- and sex-matched homozygous *IL-6Rα*^fl/fl^ mice served as wild-type (WT) controls and received the same tamoxifen treatment. Mice underwent ICH surgery 30 days after tamoxifen administration, and genotyping was validated as previously described[36]. Both *IL-6Rα ^MG-KO^* and WT mice were viable, fertile, and exhibited no gross physical or behavioral abnormalities. All animals were maintained on a 12-hour light/12-hour dark cycle to preserve their natural circadian rhythm, with free access to food and water. The animal room was kept at normal temperature and humidity. All animals were acclimatized to the housing environment for one week before undergoing any surgical procedures, and food was withheld for 8 h prior to surgery. The animals were randomly allocated to the respective treatment groups, ensuring a minimum sample size of *n* = 8 per group, as illustrated in **Fig. S1**, in accordance with rigorous experimental design standards. For *in vitro* experiments, neonatal C57BL/6J and *Cirbp*^−/−^ mice (0–13 days old) were used.

### Establishment of ICH in SD Rats, Cirbp^-/-^, IL-6Rα ^MG-KO^ and C57BL/6J mice

The procedure for inducing ICH in SD rats was performed as described in our previous study [20]. Briefly, the sham group received saline injections, and rats were housed under SPF conditions with controlled temperature and free access to food and water post-recovery. Successful ICH modeling was indicated by motor activity changes, such as circling to the right. For *Cirbp^-/-^, IL-6Rα ^MG-KO^* and C57BL/6J mice, ICH was induced following anesthesia with 1.5 % isoflurane in a 30 % O_2_/68.5 % N_2_O mixture. Mice were placed in a stereotaxic frame, ensuring proper alignment of the skull. A 1 cm midline scalp incision exposed the bregma and lambda, with adjustments made to level the skull (height difference < 0.03 mm). A small hole (1.1 mm diameter) was drilled 0.4 mm anterior to the bregma and 2 mm lateral to the midline, avoiding damage to the dura mater. A microsyringe was preloaded with collagenase IV, and 1.2 U/kg was injected at a depth of 3.5 mm from the dura, using an automatic injector at a rate of 0.1 μL/min. After injection, the needle was left in place for 5 min before partially withdrawing (1.75 mm) for another 5 min, then fully withdrawn. The incision was disinfected with iodine and sutured. Sham group mice underwent the same procedure without injection of collagenase IV. Mice were placed on a heating pad post-surgery until fully recovered and then returned to housing with normal food and water. For the ICH group, a gradient of collagenase IV (0.6 U/kg, 1.2 U/kg, and 2.4 U/kg) was injected, with 1.2 U/kg used for subsequent experiments. The ICH + TCC group received an intraperitoneal injection of 10 mg/kg Tat-CIRP-CMA (TCC) within 2 h of collagenase IV injection.

### Autologous blood injection ICH model

For the sham surgery groups (both wild-type and *IL-6Rα^MG-KO^*mice), a craniotomy was performed, a hole was drilled, but no liquid was injected. In the ICH groups, 30 μL of red blood cell suspension was injected into the brain following the same craniotomy procedure.

### Human sample collection and processing

This investigation recruited 51 patients with ICH and 37 age-matched healthy subjects from June 2023 to December 2024. A formal sample size calculation was performed using PASS software (version 16.0, NCSS, Kaysville, UT, USA) to ensure statistical robustness. Based on a two-sided α of 0.05, a statistical power (1–β) of 90 %, and a large anticipated effect size (Cohen’s d ≥ 0.8) based on the study assumption and previous studies, the minimum sample size per group was at least 23. The final sample included in the study was determined to be sufficient to meet the statistical power requirement and ensure the reliability of the results. This study was approved by the Medical Ethics Committee of Henan Provincial People's Hospital with written informed consent from ICH patients or age-matched healthy subjects (Ethics approval number: 2023–105) and Affiliated Hospital of Nantong University approval No. [Nantong 2022-K181-01]. The investigation was accomplished according to the guidelines of the Helsinki Declaration. First, patients with clinical manifestations suggestive of ICH were identified, and the diagnosis was subsequently confirmed by CT imaging. Then, the blood samples from all patients were collected before any therapeutic medication. Approximately 4–5 mL of peripheral blood samples were obtained in EDTA-K2 anticoagulant tubes, lysed in TRIzol regents, and then stored in a −80 °C refrigerator. Inclusion criteria: Eligible patients were those diagnosed with ICH via CT imaging, aged 36 to 80, and who underwent CT within 24 h of symptom onset. Written informed consent was required from both the patient and their family. Exclusion criteria included patients with severe complications upon admission, a history of cerebrovascular diseases (e.g., head trauma, vascular malformations, or brain tumors), coagulopathy, significant hepatic or renal impairment, or inability to cooperate with examinations. Detailed patient characteristics, including age, gender, comorbidities, the precise location of the ICH, and the timing of blood collection post-ICH (measured in hours/days), are comprehensively documented in Table S1.

### Single cell sequencing (scRNA-seq) analysis

Striatal samples collected for scRNA-seq analysis three days after model induction, with rats anesthetized using isoflurane inhalation. Each group had three brain tissue samples, which were precisely cut into 0.3 cm × 0.3 cm pieces on ice. The collection, handling, and preparation of the samples were meticulously conducted, ensuring accurate sectioning and immediate shipment to Shanghai Genechem Co., LTD for scRNA-seq analysis. Cells were captured, and a 3′ end library was constructed and RNA sequencing was performed on the NovaSeq 6000 platform (Illumina, San Diego, CA, USA). Quality control was conducted using FASTP and BD Rhapsody. The Rhapsody bioinformatics tool was used to process Fastq files, extract UMI sequences, and analyze cell quality and gene expression profiles. Data normalization and batch effect correction were performed using the Seurat package, and clusters were visualized with UMAP. Cells were annotated using marker genes from the CellMarker database (http://biocc.hrbmu.edu.cn/CellMarker/).

### Overexpression plasmid construction

The overexpression plasmid for IL-6R was constructed by GenScript Biotech using a pcDNA3.1(+)-3xFLAG-P2A-EGFP vector with a broad-spectrum promoter, non-fusion EGFP fluorescence, and Neo (G418) resistance. The final construct was pcDNA3.1(+)-IL6r-3xFLAG-P2A-EGFP, and a control plasmid (pcDNA3.1(+)-3xFLAG-P2A-EGFP) was also generated.

### Transfection of HEK 293 T cells

HEK 293 T cells were cultured in six-well plates to 70–80 % confluence. Cells were divided into control and test groups. For transfection, solution A (plasmid diluted in Opti-MEM) and solution B (Lipofectamine 2000, Life Technologies, USA, diluted in Opti-MEM) were mixed at a 1:2 plasmid-to-lipofectamine ratio. After 6 h, the medium was replaced with DMEM, and GFP fluorescence was observed 24–48 h post-transfection to assess efficiency. Cells were divided into PBS, IL-6R, and CIRP (1 μg/mL for 24 h) groups for further analysis.

### Co-immunoprecipitation (Co-IP)

Cells were washed with PBS and lysed in a buffer containing 20 mM Tris-HCl, 137 mM NaCl, 3 mM KCl, 10 % glycerol, 1 mM EDTA, and 0.3 % Triton X-100. Lysates were incubated with Protein A/G magnetic beads (MCE HY-K0202, 1 mL) and primary antibodies against IgG and CIRP (Proteintech, 10209–2-AP) at 4 °C overnight. The beads were washed twice with lysis buffer to remove unbound proteins. Bound proteins were eluted by boiling the beads in sample buffer at 95°C for 3 min and subjected to Western blot analysis.

### Macromolecular docking and peptide design

Drawing upon the differential expression of the gene *Cirbp* and the pivotal IL-6-JAK-STAT pathway subsequent to brain hemorrhage, we conducted macromolecular docking between CIRP (UniProtKB ID: Q14011) and IL-6R (UniProtKB ID: P08887) utilizing the GRAMM-X software. This approach aimed to predict the binding interface between CIRP and the IL-6Rα receptor, following established methodologies described in prior research[35]. We designed two peptides (Tat-CIRP-CMA, TCC): Tat is an 11-amino acid transmembrane sequence; CIRP is a 15-amino acid sequence from positions 111–125 with high affinity for both MD2 and IL-6Rα; CMA is a 14-amino acid signal peptide sequence targeting the proteasome. The peptide sequence is YGRKKRRQRRR-GRGFSRGGGDRGYGG-KFERQKILDQRFFE (Heifei KS-V Peptide Biotechnology, China). To further investigate peptides targeting IL-6Rα with high specificity, we synthesized a new peptide (Tat-CIRP, TC): Tat is an 11-amino acid transmembrane sequence; CIRP is a 15-amino acid sequence from positions 127–141 with high affinity for IL-6Rα; peptide sequence: YGRKKRRQRRRRFESRSGGYGGSRD (Heifei KS-V Peptide Biotechnology, China). Both peptides were dissolved in 0.9 % saline at a concentration of 10 mg/kg for intraperitoneal injection. The treatment group received the peptide two hours post-hemorrhage, while the ICH control group received saline injections. Three days after TCC intervention in rats, samples were sent for single-cell sequencing. To validate the effects of TCC and TC *in vitro*, microglial cells were pre-treated with the peptides (10 μM) for 0.5 h, followed by stimulation with recombinant mouse CIRP (rmCIRP, 1 μg/mL) for 24 h to establish an inflammation model.

### Surface plasmon resonance (SPR)

SPR technology, supported by Sino Biological, was employed to measure the binding affinities between human IL-6Rα, CIRBP, and TCC. Human IL-6Rα was immobilized on a CM5 chip, and its interaction with human CIRBP was first analyzed to determine binding affinity. Subsequently, the affinity of IL-6Rα for TCC was measured under similar conditions. In a competitive inhibition assay, TCC, at 100 times its affinity constant, was used to saturate IL-6Rα, testing whether IL-6Rα could still bind CIRBP in the presence of high levels of TCC.

### Enzyme-linked immunosorbent assay (ELISA) Detection

ELISA was used to quantify CIRP, MD2, IL-6, and TNF-α concentrations in blood and cerebrospinal fluid samples. Samples were processed and stored under standardized conditions to preserve protein integrity. Each target protein was measured using commercial ELISA kits, following the manufacturer’s instructions: Mouse CIRBP ELISA Kit (CSB-EL005440MO, CUSABIO), Mouse TNF-α ELISA Kit (EK282, Lianke Bio), Mouse IL-6 ELISA Kit (CSB-E04639m, CUSABIO), and Rat TNF-α ELISA Kit (EK382, Lianke Bio).

### Behavioral assessments

Behavioral tests were conducted on rats and mice at 1, 2, 3, 4, 5, 7, 14, 21, 22, 23, 24, and 28 days post-ICH.

### Modified neurological severity score (mNSS)

The mNSS assessed neurological function, combining motor, sensory, and reflex tests. Scores ranged from 0 (normal) to 18, with higher scores indicating more severe injury. Severity was classified as follows: 13–18 (severe damage), 7–12 (moderate damage), and 1–6 (mild damage).

### Foot fault/grid walking test

This test evaluated sensorimotor function on days 1, 3, 5, 7, 14, and 28 post-ICH. Rats walked on a grid (50 cm × 40 cm × 30 cm), and forelimb or hindlimb missteps were recorded. After a 3-day pre-training period, the number of missteps and total steps taken during a 1-minute trial were recorded. The misstep rate was calculated as (missteps / total steps) × 100 %.

### Rotarod test

Mice were acclimated to the Rotarod apparatus for 3 days pre-surgery. During the test, the Rotarod speed increased from 4 to 40 rpm over 5 min. Mice were trained 3–4 times, with the last day’s value as the baseline. Each mouse underwent three trials, with a 15-minute interval between trials. If the mouse fell or clung to the rod for one full revolution without moving, the time was recorded, with a maximum time of 5 min. The average time across trials was used to assess motor function, with lower times indicating more severe damage.

### Corner turn test

The corner turn test evaluated sensorimotor asymmetry. Rats were placed between two 30-degree angled boards. Upon entering the corner, they would turn towards the entering edge. Each rat underwent 10 trials, with 1-minute intervals between trials.

### Water maze

The water maze was used to assess learning and memory. Pre-surgery, animals were trained for 3 days to locate a hidden platform. Post-surgery, testing occurred over 4 consecutive days, recording the time taken to find the platform and swim trajectory. On the final day, the platform was removed, and the time spent in the target quadrant within 1 min was recorded to assess memory retention.

### Nissl, hematoxylin and eosin (HE) staining

Tissue sections were dehydrated sequentially in 100 %, 95 %, 90 %, 80 %, 70 %, and 50 % ethanol for 1–2 min each, followed by two rinses in distilled water for 30 s. For HE staining, sections were stained with hematoxylin (Beyotime, Shanghai, China) for < 10 min to visualize nuclei, followed by rinsing and differentiation for 30 s to 3 min. After rinsing with distilled water, eosin staining was performed for 1 min. For Nissl staining, sections were immersed in Nissl solution for 10 min. Dehydration was performed sequentially with 50 %, 70 %, 80 %, 90 %, 95 %, and 100 % ethanol for 1–2 min each, followed by xylene for transparency (3 times, 2 min each). Sections were sealed with neutral balsam.

### Fast blue staining

The staining solution was preheated in a 60 °C oven for 30 min to 2 h. Brain slices were passed through distilled water three times, then immersed in the preheated staining solution for 2–3 h. Following staining, sections were rinsed in 95 % ethanol for 1–2 min, then in distilled water for 1 min. After soaking in 0.05 % lithium carbonate solution for 1 min, slices were differentiated by immersion in 70 % ethanol, followed by observation under a microscope to distinguish gray and white matter. Rapid dehydration was performed using anhydrous ethanol (3 times), followed by xylene (3 times, 1–2 min each). Sections were sealed with neutral balsam, and images were captured under a microscope (Leica, GER), with white matter myelin appearing as deep blue.

### Immunofluorescence

Brain tissues were fixed in paraformaldehyde, dehydrated, and sectioned at 30 µm using a microtome (CM1900UV, Leica, Germany). Sections were floated onto glass slides and left overnight at room temperature. For cultured cells, after discarding the medium, cells were washed twice with PBS, fixed with precooled 4 % PFA at 4 °C for 10 min, and washed three times with PBS (10 min each) on a shaker. Cells were permeabilized with 0.3 % Triton X-100 in PBS for 20 min, blocked with 5 % donkey serum for 1 h, and incubated overnight at 4 °C with primary antibodies (Iba-1, Rabbit polyclonal, Wako; NeuN, Millipore; GFAP, CST; MBP, Abcam; SMI32, Millipore). The next day, sections were brought to room temperature, washed three times with PBST (PBS + Tween-20), and incubated with secondary antibodies for 1 h in the dark. After three more PBST washes, samples were mounted with DAPI-containing medium and sealed. Fluorescence was visualized using a DM4000B microscope and images were captured with a Leica TCS SP8 confocal microscope.

### Primary neuron and microglia culture and phenotyping induction

Primary cortical neurons were isolated from embryonic day 16–18 (E16–E18) C57BL/6 mouse embryos as previously described, and cultured in Neurobasal medium supplemented with B27 and GlutaMAX (Gibco).Primary microglial cells were isolated from the cortices of neonatal (day 1) C57BL/6J mice. After disinfection with 75 % ethanol, the skin and skull were removed, and the brains were placed in ice-cold HBSS. The meninges were peeled off, the cortex was collected, and the hippocampus was discarded. Tissues were digested with 0.25 % trypsin at 37 °C for 12 min, centrifuged at 1200 rpm for 5 min, and resuspended in complete medium. The suspension was transferred to a T75 flask with 15 mL of complete medium. After 14 days, microglial cells were harvested by shaking the flask at 270–280 rpm for 30 min, and the supernatant was collected. To induce phenotyping, microglial cells were treated with LPS (100 ng/mL) and IFN-γ (20 ng/mL) for 48 h to induce the M1 phenotype, or IL-4 (20 ng/mL) for 48 h to induce the M2 phenotype. CIRP (1 μg/mL for 24 h) was added to the microglia, which were divided into PBS, CIRP, and CIRP + TCC groups. TCC (10 μM) was administered for 48 h.

### Oxygen-Glucose deprivation (OGD) model and CIRP,TCC treatments

To mimic ischemic conditions, neurons at DIV7 were subjected to OGD. Briefly, culture medium was replaced with glucose-free DMEM (Gibco), and neurons were incubated in a hypoxic chamber (1 % O_2_, 5 % CO_2_, 94 % N_2_) at 37℃ for 4 h, followed by reoxygenation in normal culture conditions for 24 h. The supernatants were collected for subsequent CIRP quantification. Recombinant CIRP protein was used to stimulate microglia at a concentration of 1 μg/mL for 24 h. For intervention experiments, microglia were pre-treated with TCC for 1 h prior to CIRP stimulation and maintained in the presence of TCC throughout the 24-hour stimulation.

### Cell Counting Kit-8 (CCK-8) assay

Microglia were exposed to 1, 10 100 μM concentrations of TCC or TC and PBS respectively for 24 h, and cell viability was measured. Neurons were co-cultured with microglial conditioned media for 24 h. Cell viability was assessed using the Cell Counting Kit-8 (C0038, Beyotime) according to the manufacturer’s protocol. Absorbance at 450 nm was measured to determine cell viability.

### RNA extraction and quantitative real-time PCR (qRT-PCR)

RNA was extracted using TRIzol reagent (Invitrogen, 210901). RNA was reverse transcribed into cDNA using Novagen's R323-01 100RXN. qRT-PCR was performed to determine the mRNA levels of *Nos2, Tnf, Rela, Mcr1, Arg1, Cirbp, Il6a and Il6*. The primer sequences used are as listed in the Table S2. qRT-PCR was conducted using the StepOne Real-Time PCR System (Life Technologies, USA). The qRT-PCR protocol included an initial denaturation at 95 °C for 5 min, followed by 40 cycles of 60 °C for 30 s and 72 °C for 10 s.

### Flow cytometry

On the third day post-ICH, microglial phagocytosis of RBCs was analyzed by flow cytometry (CytoFLEX Flow Cytometer (PN: B49006AE, Beckman Coulter)). Mouse brains were perfused with cold PBS, and the right hemisphere was isolated, minced, and incubated with enzyme solutions. The tissue was processed into a single-cell suspension, filtered, and stained with specific antibodies and viability dyes. After washing and fixing with PFA, samples were stored at 4 °C in the dark and analyzed within 72 h.

### Western blot

Brain tissue adjacent to the hemorrhagic striatum was homogenized in lysis buffer with protease inhibitors. Cells were washed twice with PBS, lysed, and sonicated. After a 30-minute rest to ensure thorough lysis, the homogenate was centrifuged at 12,000 rpm at 4 °C for 15 min. The supernatant was collected for protein quantification using the BCA method. Samples were prepared at a concentration of 2 μg/μL, and 15 μL per well was loaded for gel electrophoresis. Proteins were transferred to membranes using a wet transfer method and blocked with 5 % non-fat milk. Primary antibodies used were CIRBP (Proteintech, 10209–2-AP), NF-κB (CST, 6956S), HMGB1 (CST, 6893S), Phospho-NF-κB p65 (CST, 3033 T), STAT3 (CST, 4904 T), Phospho-STAT3 (CST, 9145 T), IL-6R (Proteintech, 23457–1-AP), CD206 (CST, 24595 T), and CD16 (Novus, NBP2-42228). Membranes were incubated with primary antibodies overnight at 4 °C, followed by incubation with secondary antibodies (GAR-HRP, BIOSHARP; GAM-HRP, Jackson) at room temperature for 2 h. Membranes were washed three times with TBST and developed using a chemiluminescent substrate.

### NIH stroke scale (NIHSS) Evaluation

Patients' neurological deficits were evaluated using the NIHSS. This standardized scale was used to quantify stroke severity by assessing various aspects of neurological function, including level of consciousness, visual fields, motor skills, sensory abilities, and language.

### Computed tomography (CT) scan

CT scans were performed using a high-speed scanner (GE Medical System) for all patients to confirm ICH diagnosis and assess hemorrhage location and extent. Initial CT imaging was conducted within 24 h of admission to monitor hemorrhage progression.

### Data analysis

All statistical analyses were performed using GraphPad Prism version 8.0. Data are expressed as mean ± standard deviation (SD). Depending on the distribution, data were analyzed using parametric or non-parametric tests. After assessing normality, comparisons between two groups were made using independent samples t-tests; comparisons among more than two groups were conducted using one-way ANOVA with post-hoc Tukey's tests. Two-way ANOVA was used when there were two factors jointly influencing the observed variable and the data were consistent with normal distribution and chi-square. *P* < 0.05 were considered statistically significant.

## Results

### Peripheral blood expression of CIRP is elevated and positively correlative with infarct volume in ICH patients

**Fig. 1:**
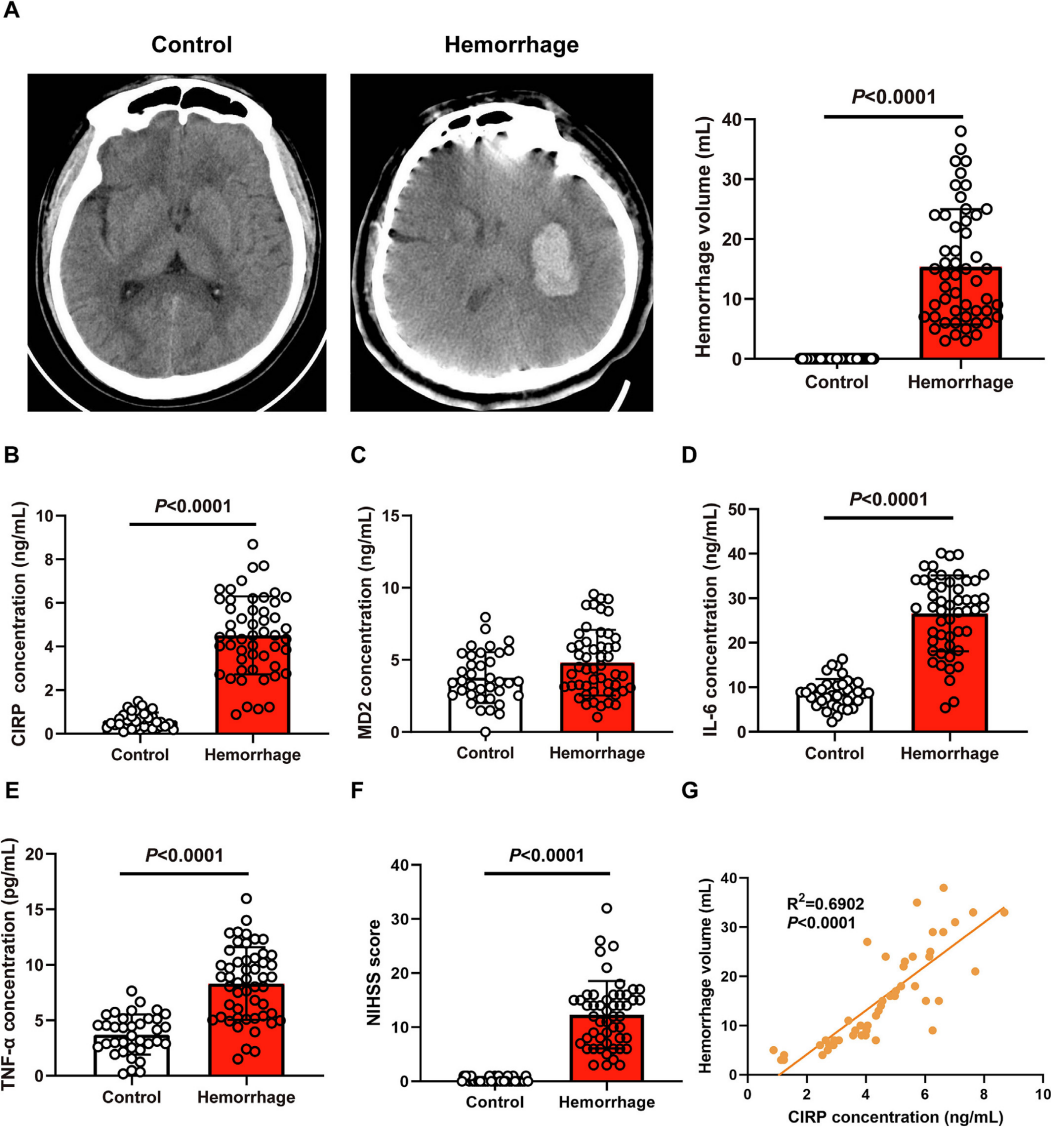
Peripheral blood expression of cold-inducible RNA-binding protein (CIRP) is elevated and positively correlated with infarct volume in intracerebral hemorrhage (ICH) patients **A** Representative Computed Tomography (CT) scan of a patient with ICH showing a hyperdense (bright) elliptical lesion in the right cerebral hemisphere, indicating a hemorrhagic area. Surrounding the hemorrhage is a hypodense (darker) region suggesting edema. Mild displacement of brain sulci and ventricular structures can also be observed due to hemorrhage-related pressure. Hemorrhage volume was calculated for each patient. **B-E** Quantification of CIRP, Myeloid Differentiation Protein 2 (MD2), interleukin-6 (IL-6) and tumor necrosis factor-alpha (TNF-α) levels in serum were measured using enzyme-linked immunosorbent assay (ELISA). *n* = 37–51. **F** National Institutes of Health Stroke Scale (NIHSS) scores for patients. *n* = 37–51. **G** Correlation analysis between CIRP levels in blood and hemorrhage volume in ICH patients, revealing a statistically significant positive association, suggesting a potential link between CIRP expression and ICH severity.

As part of our clinical analysis, the baseline characteristics of the enrolled ICH patients are summarized in **Table. S1**. As shown in Fig. 1A, the CT scan presents an axial view of the brain, where a hyperdense (bright) elliptical lesion is visible in the right cerebral hemisphere. This hyperdense region represents a hemorrhagic lesion, caused by intracerebral bleeding. Surrounding the hemorrhagic area, there appears to be a region of low density or mild edema, potentially related to compression of adjacent brain tissue or secondary swelling. Additionally, due to the pressure from the hemorrhage, there is a slight shift in the nearby brain sulci and ventricular structures. Quantitative analysis revealed that the ICH volume in the ICH group was markedly higher than that in the control group, consistent with the expected pathological features. In Fig. 1**B-E**, the expression levels of CIRP, IL-6, and TNF-α in serum were significantly higher in ICH patients compared to the control group, as quantified by ELISA. While MD2 levels were not statistically different, a trend toward increased expression was observed. Additionally, in Fig. 1F, the NIHSS score was elevated in the hemorrhage group, indicating more severe neurological deficits in patients. Correlation analysis further demonstrated a positive association between CIRP transcript levels in blood and ICH volume in stroke patients, with a statistically significant correlation, suggesting that CIRP expression may correlate with stroke severity (Fig. 1G).

### Single-cell sequencing identifies genes and pathways associated with microglia following ICH

**Fig. 2:**
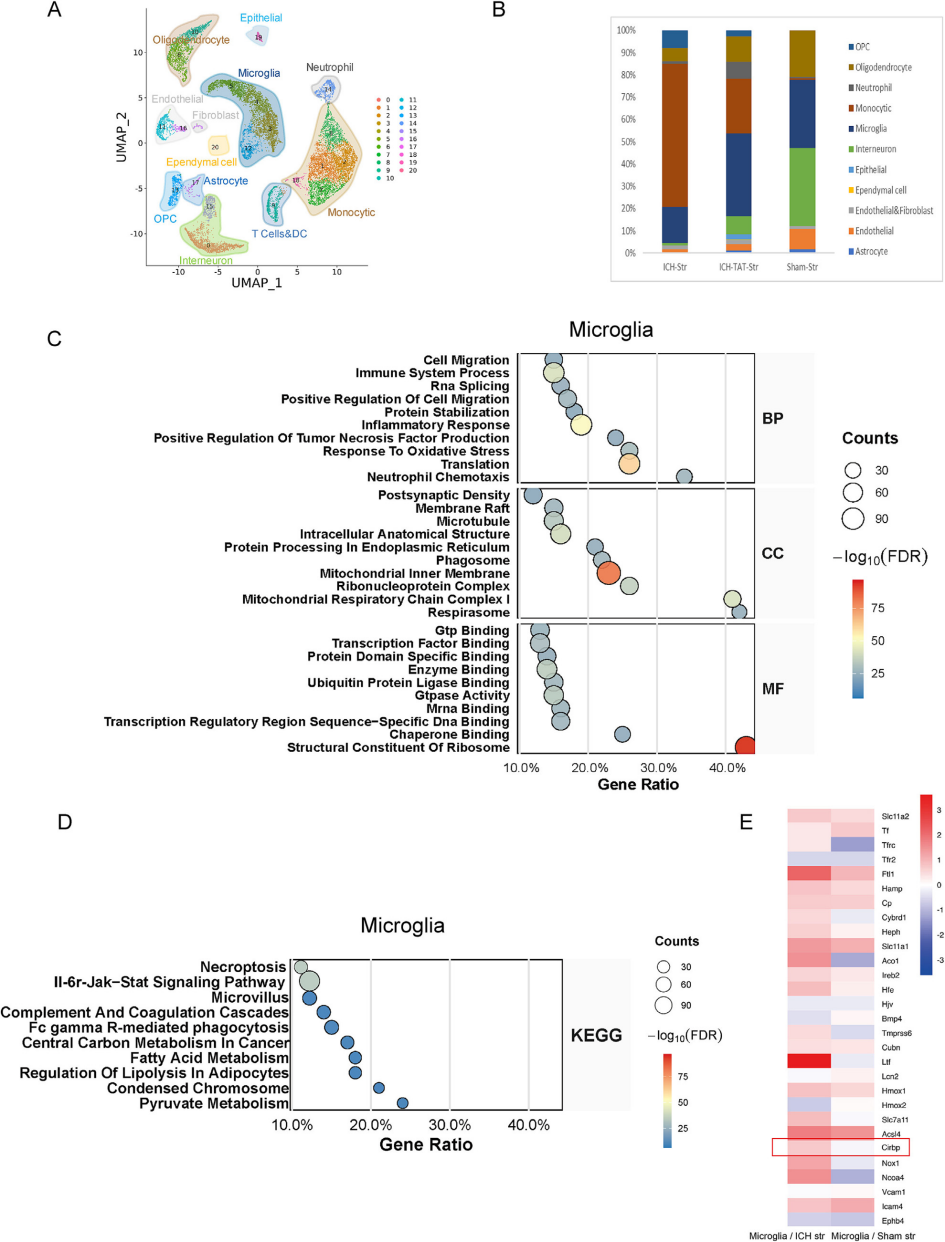
Single-cell sequencing reveals genes and pathways linked to microglia following ICH **A** Uniform Manifold Approximation and Projection (UMAP) plot showing the distribution and clustering of major brain cell types identified from single-cell RNA sequencing of striatal tissue in sham and ICH groups. **B** Bar plot displaying the relative proportions of each cell type across clusters. **C** Gene Ontology (GO) enrichment analysis of differentially expressed genes (DEGs) in microglia, highlighting the top 10 significantly enriched biological processes, molecular functions and cellular component. **D** Kyoto Encyclopedia of Genes and Genomes (KEGG) pathway analysis of key pathways in microglia from sham and ICH groups. Bubble plots are used to visualize the results of GO and KEGG enrichment analyses, respectively. The y-axis displays the top 10 significantly enriched terms or pathways, while the x-axis represents the gene ratio (the proportion of differentially expressed genes involved in each term). The size of each bubble corresponds to the number of enriched genes, and the color gradient reflects the statistical significance (adjusted p-value), with redder colors indicating higher significance. **E** Heatmap illustrating changes in *Cirbp* expressionof microglia in sham and ICH groups.

Subsequently, we performed single-cell RNA sequencing on striatal tissues from the ipsilateral (hemorrhagic) hemisphere of rats 3 days after ICH induction. Using this approach, we identified key genes and pathways involved in microglial activity post-ICH. As shown in Fig. 2A, Uniform Manifold Approximation and Projection (UMAP) was applied to visualize the cellular heterogeneity in brain tissue from sham and ICH groups. Distinct clusters corresponding to major brain cell types—including microglia, astrocytes, interneurons, oligodendrocytes, endothelial cells, and others—were clearly separated, indicating robust cell-type classification across samples. Bar graphs showed the distribution of each cell type across all clusters (Fig. 2B). Notably, the proportion of immune cells was markedly changed in the ICH group compared to the sham group, suggesting a immune response following ICH. Conversely, proportions of interneurons and oligodendrocytes were relatively reduced, possibly reflecting injury-associated loss or dysfunction. A heatmap was generated to visualize the expression changes of *Cirbp* and other inflammation-related genes in microglia and monocytes between sham and ICH conditions (**Fig. S2**A). It showed that CIRP expression in microglia fluctuates significantly more than in monocytes following ICH. In Fig. 2C, the top 10 enriched Gene Ontology (GO) terms in microglia, as referenced in [37], demonstrated a strong enrichment of differentially expressed genes (DEGs) primarily involved in essential biological processes and molecular functions. These included pathways related to protein translation (e.g., “structural constituent of ribosome,” “ribosomal subunit”), indicating enhanced biosynthetic activity; inflammatory and immune responses (e.g., “inflammatory response,” “immune system process,” “response to cytokine”), reflecting the activation of microglia as innate immune effector cells during ICH; and mitochondrial function and bioenergetics (e.g., “mitochondrial inner membrane,” “oxidative phosphorylation”), suggesting increased metabolic demand and potential mitochondrial reprogramming in response to the injury. Collectively, these enriched GO terms highlight the multifaceted reactivity of microglia following ICH, characterized by heightened immune surveillance, upregulated protein synthesis machinery, and altered mitochondrial activity, all of which contribute to the neuroinflammatory microenvironment. KEGG pathway analysis, as referenced in [38], revealed a selective and significant enrichment of the IL-6R–JAK–STAT signaling pathway in microglia from the ICH group compared to the sham group (Fig. 2D). This pathway plays a pivotal role in mediating inflammatory signaling, cellular proliferation, and immune modulation. The enrichment of this pathway suggests that microglia, upon sensing hemorrhagic injury, may engage the IL-6r–JAK–STAT signaling cascade in response to elevated interleukin-6 levels, potentially leading to the transcription of pro-inflammatory genes and contributing to the neuroinflammatory process. The significant enrichment of this pathway highlights its potential involvement in microglial activation following intracerebral hemorrhage and supports its relevance as a therapeutic target for modulating post-ICH inflammation.

In addition, all enrichment results comparing the sham and ICH groups have been systematically reorganized and categorized. For GO analysis, the data have been reclassified by GO category, term name, total number of genes in the gene set, number of overlapping genes, p-value, and false discovery rate (FDR). A similar classification was performed for KEGG pathway analysis. All detailed results are provided in **Table S3.** A heatmap was generated to assess the expression of *Cirbp* in microglia (Fig. 2E). In the ICH group, *Cirbp* expression was substantially upregulated in microglia, implicating *Cirbp* as a potential regulator of microglial activation and inflammatory signaling in the ICH microenvironment. The complete heatmap assessing the expression of *Cirbp* across different brain cell types in **Fig. S2**B. Further analysis revealed that differentially expressed genes (DEGs) in monocytes from the ICH group were significantly enriched in several biological processes and functional pathways. Notably, these DEGs were associated with myelin sheath organization, immune system processes, and lysosomal function, suggesting that monocytes may be involved not only in immune activation but also in modulating phagocytosis and myelin remodeling after ICH (**Fig. S2**C**)**. Notably, the genes displayed in the heatmap were selected based on their relevance to inflammation, oxidative stress, including *Cirbp*, which was examined in patient samples. A more comprehensive list of inflammation-related genes is provided in **Table S4.**

### *Validation of IL-6R-CIRP interaction and STAT3 effect, plus design and verification* of peptides targeting IL-6R and CIRP

**Fig. 3:**
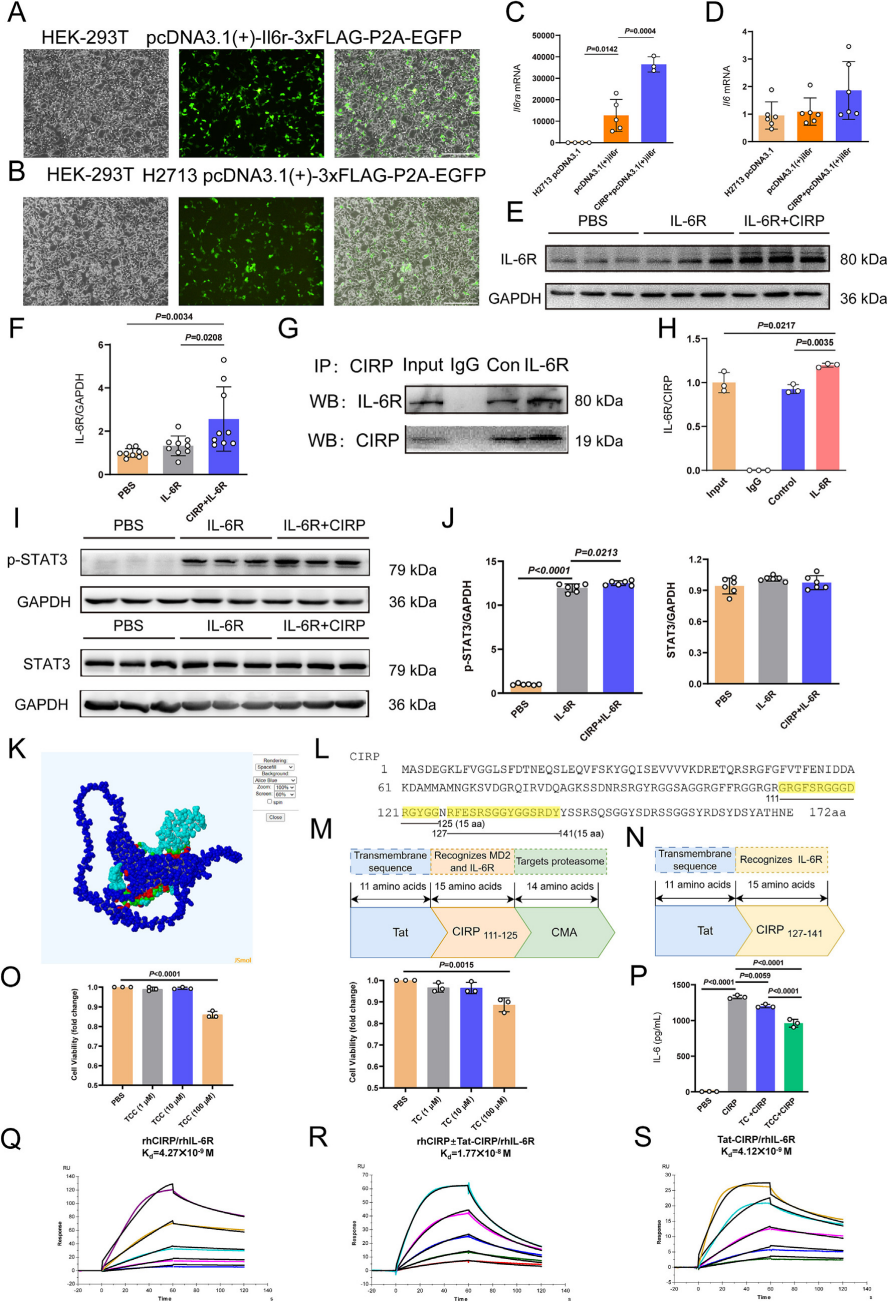
Validation of interleukin-6 receptor (IL-6R)- CIRP interaction and signal transducer and activator of transcription 3 (STAT3) effect, plus design and verification of peptides targeting IL-6R and CIRP **A-B** Successful construction of IL-6R-overexpressing human embryonic kidney 293 T (HEK-293 T) cells, confirmed by stable green fluorescent protein (GFP) fluorescence; scale bar = 100 μm. **C–D** Quantitative reverse transcription polymerase chain reaction (qRT-PCR) results showing increased Il6ra and Il6 mRNA expression in HEK-293 T cells after CIRP stimulation. *n* = 3–6. **E, F** Western blot analysis of IL-6R protein expression in IL-6R-overexpressing cells following CIRP stimulation, with statistical analysis. *n* = 9. **G, H** Co-immunoprecipitation confirming the direct interaction between CIRP and IL-6R, with statistical analysis. *n* = 3. **I, J** Western blot analysis of phosphorylated STAT3 (p-STAT3) and total STAT3 activation following CIRP and IL-6R interaction, and after Tat-CIRP-CMA (TCC) treatment. *n* = 6. **K** Macromolecular docking of CIRP and IL-6R alpha subunit (IL-6Rα) using GRAMM-X, followed by visualization of the interaction with PDBePISA. The “Interfacing residues” section lists the involved residues, types of chemical bonds (e.g., hydrogen bonds, salt bridges), and the ratio of the buried surface area. The red region marks the tight binding site between CIRP and IL-6Rα. **L** Full-length CIRP amino acid sequence and the identified tight-binding sequence. **M** Schematic of the designed peptide Tat-CIRP-CMA (TCC). **N** Schematic of the designed peptide Tat-CIRP (TC). **O** Microglia were exposed to increasing concentrations of TCC or TC for 24 h, and cell viability was measured using the Cell Counting Kit-8 (CCK-8) assay, *n* = 3. **P** Enzyme-linked immunosorbent assay (ELISA) results showing IL-6 release in cell culture supernatants following treatments with TC and TCC peptides, *n* = 3. Q**–S** Surface plasmon resonance (SPR) analysis of binding affinity between recombinant human CIRP (rhCIRP) and recombinant human IL-6R (rhIL-6R) without TCC, between IL and 6R and TCC, and between IL and 6R and CIRP in the presence of TCC. The X-axis indicates time, and the Y-axis shows response.

Based on the single-cell transcriptomic analyses presented in Fig. 2, our study revealed a relative significant enrichment of inflammation-related pathways—IL-6R–JAK–STAT signaling cascade—in microglia following ICH. The pathway-level enrichment suggests a coordinated transcriptional response associated with inflammatory activation. Concurrently, we observed increased expression of *Cirbp* in microglia, which, together with the enrichment of the IL-6R signaling pathway, points to a potential mechanistic link between CIRP and IL-6Rα in post-ICH neuroinflammation. In addition, based on existing literature, CIRP acts as a novel ligand for IL6Rα in sepsis [34].To further investigate this interaction and its downstream effects, we next sought to experimentally validate the CIRP/IL-6R signaling axis using *in vitro* approaches. We constructed an *in vitro* cell line overexpressing IL-6R and examined its interaction with CIRP. Successful overexpression of IL6R in HEK-293 T cells was confirmed by GFP fluorescence following transient transfection, as shown in Fig. 3**A-B**. To investigate the effect of CIRP on IL-6R signaling, we examined the transcriptional changes of Il6ra and Il6 in an IL-6Rα-overexpressing cell line. Following exogenous CIRP stimulation, we observed a significant upregulation of Il6ra mRNA and protein expression (Fig. 3**C,E,F**), which is consistent with our expectation based on the known physical interaction between CIRP and IL-6Rα. This finding suggests that CIRP may not only bind IL-6Rα at the protein level but also enhance its transcription, potentially amplifying downstream JAK-STAT signaling. Interestingly, *Il6* mRNA expression did not show a significant change between groups (Fig. 3D), indicating that CIRP’s modulatory effect may be more specific to the receptor level rather than the ligand itself. This differential regulation implies a possible mechanism through which CIRP potentiates IL-6R-mediated inflammatory responses.

Co-immunoprecipitation analysis further confirmed a interaction between CIRP and IL-6R (Fig. 3**G,H**). Additionally, we investigated the downstream effects of this interaction. Western blot analysis revealed that binding of CIRP to IL-6R induced phosphorylation of the STAT3, as shown in Fig. 3**I and J**. This phosphorylation suggests that CIRP/IL-6R interaction triggers activation of the STAT3 signaling pathway.

Based on the differential expression of *Cirbp* and the enrichment of the IL-6-JAK-STAT pathway following brain hemorrhage, we conducted macromolecular docking of CIRP and IL-6Rα using GRAMM-X to predict the binding sequence between these molecules (Fig. 3**K, L**). Two peptides, TCC and TC, were subsequently designed to target this interaction (Fig. 3**M, N**). To verify the effects of these peptides, we pretreated microglia with either TCC or TC (10 μM) for 0.5 h, followed by stimulation with rmCIRP (1 μg/mL) for 24 h, creating an *in vitro* model of microglial inflammation. The concentration of 10  μM in TCC or TC was selected based on results from a CCK-8 (Fig. 3O), which demonstrated that this dose did not affect microglial viability and was thus suitable for downstream functional assays. ELISA analysis of IL-6 release in the culture supernatants revealed that CIRP significantly increased IL-6 levels, whereas both peptides reduced IL-6 release, with TCC exhibiting a stronger inhibitory effect than TC (Fig. 3P). Due to its superior efficacy, we selected TCC for further experiments. Next, to confirm that TCC could inhibit the binding of CIRP and IL-6Rα, we performed SPR analysis. The results showed that rhCIRP and rhIL-6Rα bound with high affinity, with an equilibrium dissociation constant (KD) of 4.27 × 10^−9^ M (Fig. 3Q). However, in the presence of 1.5 μg/mL TCC, the binding affinity significantly decreased, with a KD of 1.77 × 10^−8^ M (Fig. 3R). Additionally, we measured the binding affinity between rhIL-6Rα and TCC alone, yielding a KD of 4.12 × 10^−9^ M (Fig. 3S**).** These results indicate that TCC effectively inhibits the binding of IL-6Rα to CIRP.

### TCC demonstrates both short- and long-term neuroprotective effects in rats following ICH

**Fig. 4:**
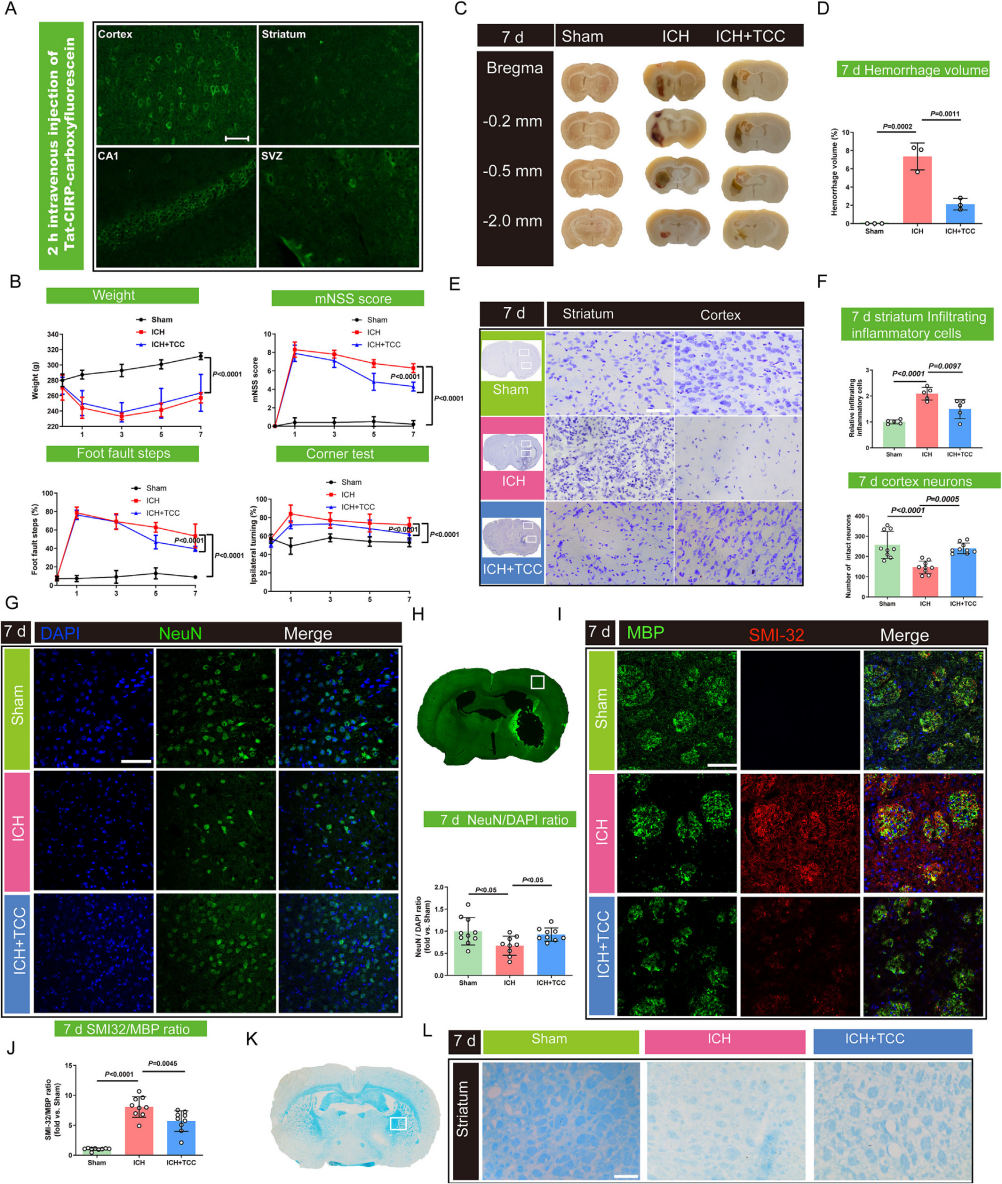
TCC improves short-term sensorimotor function, reduces hemorrhage, decreases neuronal death, and alleviates demyelination in ICH rats **A** Fluorescence distribution in the cortex, striatum, cornu ammonis 1 (CA1) region of the hippocampus, and subventricular zone (SVZ) two hours after intravenous injection of Tat-CIRP-carboxyfluorescein (10 mg/kg); scale bar = 50 μm. **B** Effects of TCC on body weight and neurological behaviors, including modified Neurological Severity Score (mNSS), foot fault test, and corner turn test in ICH rats at 1, 3, 5, and 7 days post-injury. *n* = 10. **C, D** TCC effects on coronal brain slices showing hematoma levels and calculated hemorrhage volume percentages. *n* = 3. **E, F** Nissl staining of cortical and striatal brain regions was performed to assess tissue loss and necrosis in the cortex, along with prominent infiltration of inflammatory cells in the striatum seven days after TCC administration; scale bar = 50 μm. *n* = 5–9. **G, H** Immunofluorescence analysis of cortical neurons and relative neuronal nuclei (NeuN) expression in the white box area seven days after TCC administration. *n* = 9. **I, J** Improvements in demyelination, shown by myelin basic protein (MBP) and SMI-32 (non-phosphorylated neurofilament marker) staining, with the SMI-32/MBP ratio calculated in the red box area; scale bar = 50 μm. *n* = 9. **K, L** Fast blue staining of white matter injury in the striatum, zoomed the white box area. Scale bar = 100 μm.

We further validated the neuroprotective effects of TCC in the ICH model. Schematic outline for the experimental design and groups *in vivo* and *in vitro* was shown in **Fig. S1**. As shown in Fig. 4A, two hours after intravenous administration of fluorescein-tagged TCC, the compound was widely distributed in various brain regions, including the cortex, striatum, hippocampus, and periventricular zones. TCC was particularly enriched in neurons, likely due to increased blood–brain barrier permeability following hemorrhagic injury, facilitating effective delivery of TCC to the areas of hemorrhage. In Fig. 4B, after inducing ICH by injecting collagenase IV into the striatum, the resulting hematoma compressed surrounding neural tissue, impairing body weight and sensorimotor function. Mortality rates were kept below 25 % within the first three days post-hemorrhage. TCC, administered intraperitoneally 2 h after hemorrhage, significantly improved neurological function, as shown by enhanced performance in the mNSS score and foot fault test on days 5 and 7, as well as the corner turn test on days 3 and 7. As seen in Fig. 4**C, D**, TCC treatment significantly reduced cerebral hemorrhage volume by day 7. Nissl staining (Fig. 4**E, F**) revealed a marked reduction in brain tissue loss and necrosis in the cortex, along with a significantly higher number of surviving neurons in the TCC-treated group compared to the ICH group. Additionally, in the striatal region, the ICH group exhibited extensive infiltration of inflammatory cells, which was notably attenuated following TCC intervention. Immunofluorescence further confirmed this increase in neuronal survival in the cortical region post-treatment (Fig. 4**G, H**). Additionally, myelin staining in the hemorrhagic striatum showed that TCC treatment decreased the SMI-32/MBP ratio, indicating less demyelination (Fig. 4**I, J**). Luxol fast blue staining also revealed that TCC improved myelin preservation in the hemorrhagic hemisphere, reducing white matter damage (Fig. 4**K,L**). TCC treatment led to a significant increase in body weight compared to the ICH group (**Fig. S3**A). Additionally, TCC improved sensorimotor performance, as demonstrated by better results in both the foot fault test and mNSS scores from days 14 to 28 post-hemorrhage (**Fig. S3**B, C). In the Morris water maze test, TCC-treated rats displayed enhanced learning and memory abilities. The latency to find the platform was shorter in the TCC group compared to the ICH group (**Fig. S3**D) with faster swimming speeds (**Fig. S3**E). Furthermore, the TCC group spent significantly more time in the target quadrant (**Fig. S3**F), and their swim paths in the target area increased after platform removal, indicating improved memory function (**Fig. S3**G). In addition to functional improvements, TCC treatment significantly reduced cerebral hemorrhage volume by day 28 (**Fig. S3**H-J). Immunofluorescence analysis of neurons in the cortex also revealed that TCC treatment reduced neuronal necrosis in the affected hemisphere at 28 days post-hemorrhage (**Fig. S3**K, L).

### TCC reduces CIRP release from neurons, affecting microglial activation and regulates polarization to mitigate inflammation in post-hemorrhagic rats

**Fig. 5:**
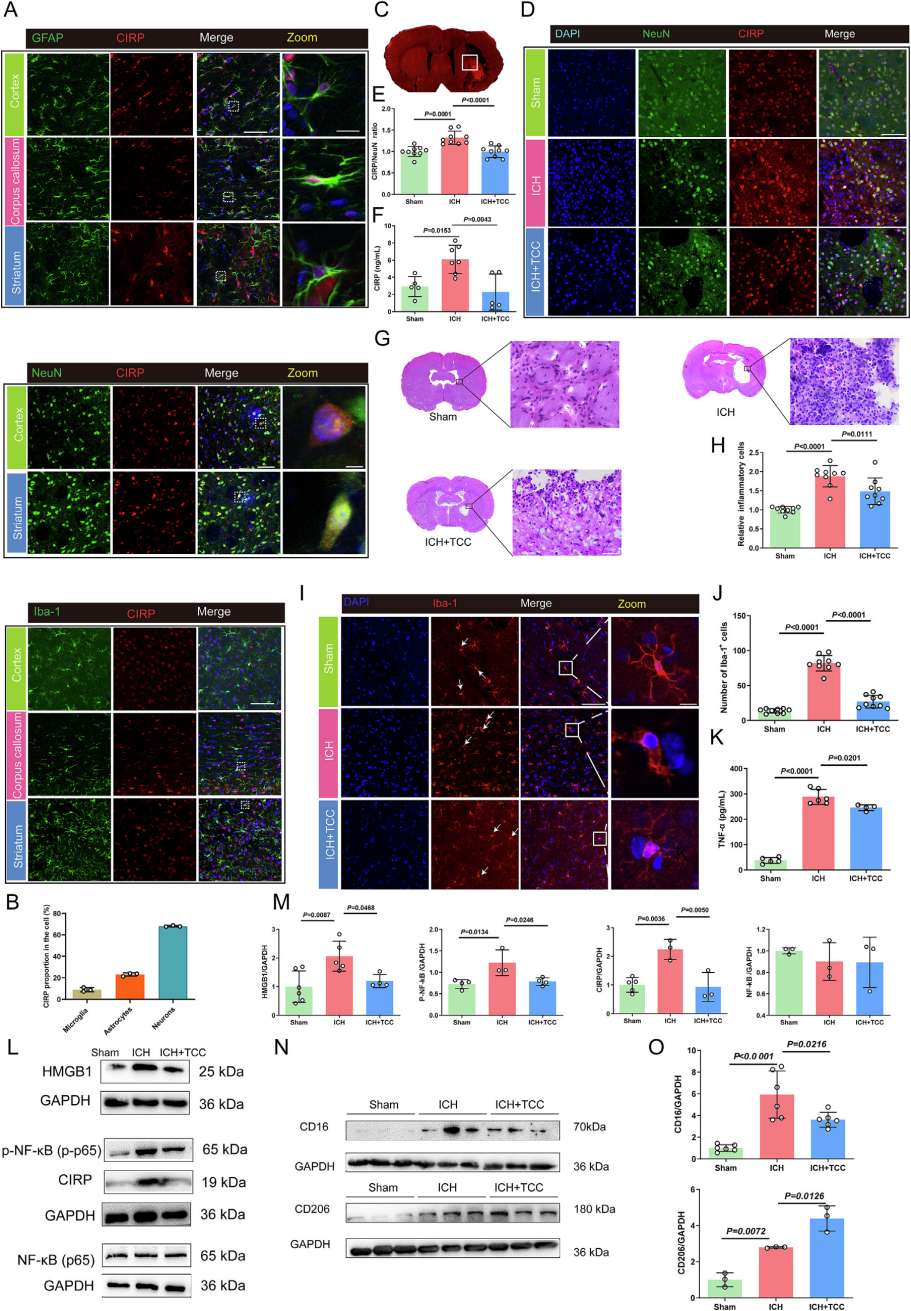
TCC reduces CIRP release from neurons, affecting microglial activation and regulates polarization to mitigate inflammation in post-hemorrhagic rats **A** Localization and statistical distribution of CIRP in astrocytes (GFAP^+^), microglia (Iba-1^+^), and neurons (NeuN-1^+^) in normal brain tissue (cortex, corpus callosum, striatum); scale bar = 50 μm, zoom scale bar = 25 μm. **B** Percentage of CIRP in different cell types. *n* = 3. C Coronal brain sections of rats showing the hemorrhagic lesion after ICH. D Immunofluorescence staining showing the change in CIRP-positive cells in striatal neurons after ICH and TCC treatment; scale bar = 50  μm. E Quantification of CIRP/NeuN double-positive cells in the striatum, *n* = 9 per group. **F** ELISA analysis of CIRP concentration in the cerebrospinal fluid from different treatment groups. *n* = 5–7. **G, H** Hematoxylin and eosin (HE) staining showing the effect of TCC on inflammatory cell infiltration in the striatum, with statistical analysis of infiltrating cell counts post-hemorrhage; scale bar = 50 μm. *n* = 9. **I, J** Immunofluorescence analysis of microglial morphology and count (ionized calcium-binding adapter molecule 1-positive cells, Iba-1^+^) in the cortex after hemorrhage; scale bar = 50 μm, zoom-in scale bar = 10 μm. *n* = 9. **K** ELISA measurement of tumor necrosis factor-alpha (TNF-α) levels in the ipsilateral striatum seven days after TCC treatment. *n* = 4–6. **L, M** Western blot analysis showing the effect of TCC on high mobility group box 1 (HMGB1), phosphorylated nuclear factor kappa B (p-NF-κB), CIRP, and total NF-κB protein levels in the striatum post-hemorrhage. *n* = 3–6. **N, O** Western blot analysis showing the effect of TCC on CD16 (M1 microglial marker) and CD206 (M2 microglial marker) expression, with corresponding statistical analysis. *n* = 3–6.

Immunofluorescence analysis revealed that under normal physiological conditions, CIRP is expressed in neurons (NeuN^+^ positive cells), astrocytes (GFAP^+^ positive cells), and microglia (Iba-1^+^ positive cells) within the cortex, corpus callosum, and striatum. The majority of CIRP co-localized with neurons, with smaller proportions co-labeled with astrocytes and microglia (Fig. 5**A, B**). Following ICH, CIRP-positive cells from the striatal neurons were observed. TCC treatment significantly reduced the amount of CIRP positive cells in neurons in the striatum (Fig. 5**C-E**). Furthermore, as depicted in Fig. 5F, TCC demonstrated a significant inhibition of the release of secretory CIRP in the cerebrospinal fluid subsequent to ICH.

The results of both single-cell sequencing and immunofluorescence above suggest that CIRP is primarily expressed by neurons before ICH, and is massively released afterward, activating immune cells including microglia. Therefore, we further investigated the effects of the CIRP-targeting peptide TCC on regulating microglia activation and inhibiting their inflammatory response. H&E staining revealed significant tissue damage in the striatal area following ICH, accompanied by extensive infiltration of inflammatory cells. TCC treatment reduced this inflammatory cell infiltration compared to the ICH group (Fig. 5**G, H**). Immunofluorescence analysis showed that TCC effectively attenuated the microglia-mediated inflammatory response post-hemorrhage. TCC-treated rats exhibited fewer microglia in the cortical areas, and their morphology shifted back to a ramified state, contrasting with the amoeboid morphology seen in ICH-induced inflammation (Fig. 5**I, J**). ELISA analysis revealed elevated TNF-α levels in the hemorrhagic striatum post-ICH, while TCC administration significantly reduced TNF-α release, indicating a mitigation of the inflammatory response (Fig. 5K). Western blot results showed that ICH increased the expression of HMGB1, CIRP, p-NF-κB proteins in the striatum, while TCC treatment reduced these pro-inflammatory markers (Fig. 5**L,M**). Further analysis revealed that TCC treatment downregulated the M1 microglial/macrophage surface marker CD16 and upregulated the M2 marker CD206, suggesting a shift from a pro-inflammatory to an anti-inflammatory phenotype (Fig. 5**N, O**).

### CIRP released by neurons after OGD activates microglia to release inflammatory cytokines, leading to neuronal necrosis, which is reversed by TCC intervention

**Fig. 6:**
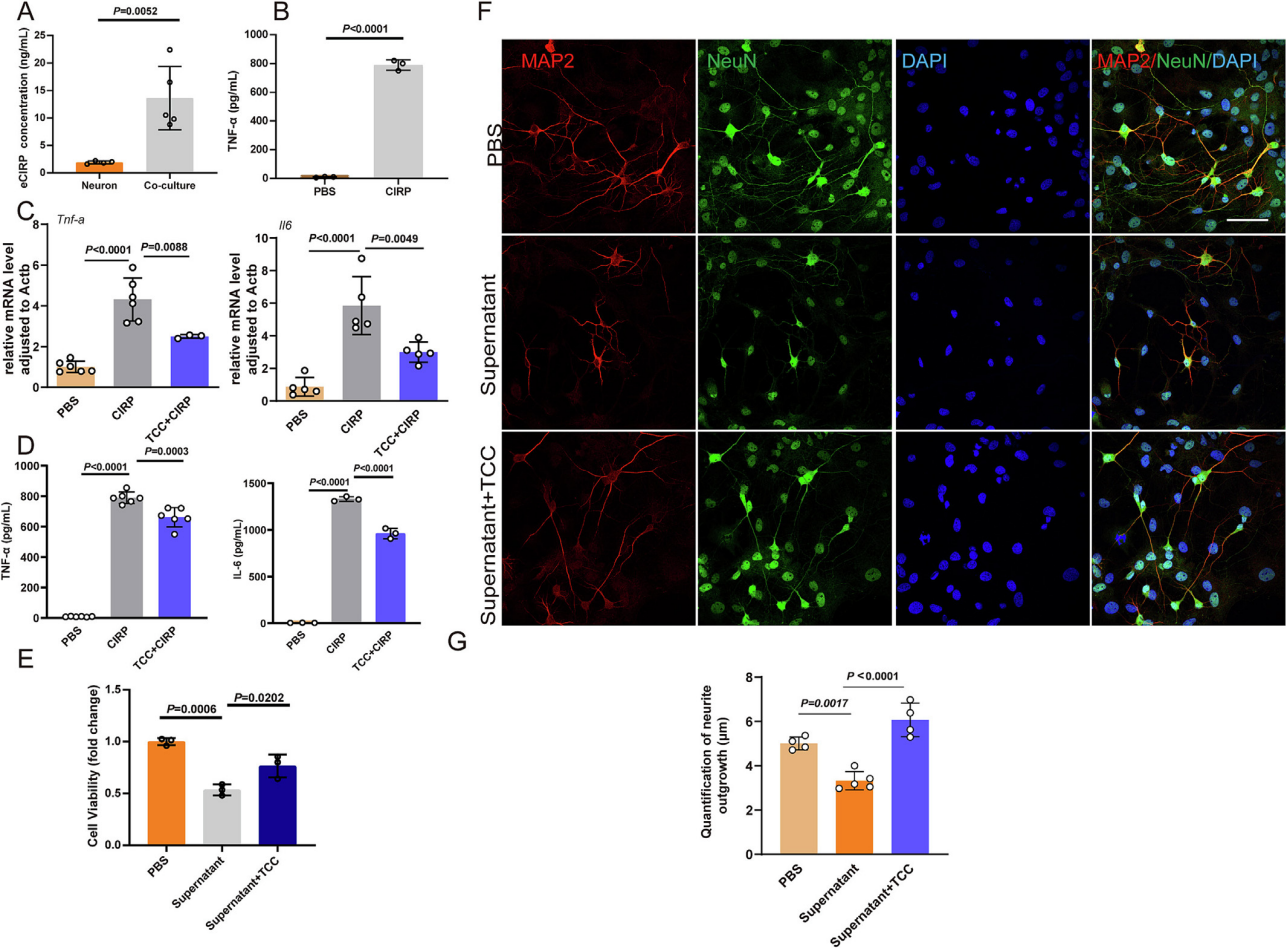
CIRP released by neurons after oxygen-glucose deprivation (OGD) activates microglia to release inflammatory cytokines, leading to neuronal necrosis, which is reversed by TCC intervention **A** ELISA analysis of CIRP levels released by neurons 24 h after OGD, *n* = 5. **B** ELISA analysis of TNF-α levels in the supernatant of microglia 24 h after rmCIRP (1 μg/mL) treatment, *n* = 3. **C** RT-qPCR analysis of TNF-α and IL-6 mRNA expression in microglia after 24-hour CIRP stimulation with or without TCC intervention, *n* = 3–6. **D** ELISA analysis of TNF-α and IL-6 levels in the supernatant of microglia after 24-hour CIRP stimulation with or without TCC intervention, *n* = 3–6. **E** Cell Counting Kit-8 (CCK-8) assay of neuronal viability after co-culture with microglial supernatant following CIRP stimulation, *n* = 3. **F-G** Immunofluorescence analysis of neuronal morphology and axonal changes after 24-hour co-culture with microglial supernatant post-CIRP stimulation, scale bar = 100 μm, *n* = 4–6.

Neurons subjected to OGD for 24 h released significantly elevated levels of CIRP, as measured by ELISA (Fig. 6A). When microglia were treated with recombinant CIRP for 24 h, the secretion of TNF-α was markedly increased (Fig. 6B). RT-qPCR analysis revealed that CIRP stimulation upregulated the transcription of pro-inflammatory cytokines TNF-α and IL-6 in microglia, while TCC intervention significantly suppressed this effect (Fig. 6C). Consistently, ELISA results showed that TCC treatment led to a moderate reduction in the levels of TNF-α and IL-6 in the supernatant of CIRP-stimulated microglia (Fig. 6D), indicating a partial attenuation of the inflammatory response (Fig. 6D). Furthermore, CCK-8 assays demonstrated that microglial supernatant following CIRP treatment impaired neuronal viability, which was partially rescued by TCC intervention (Fig. 6E). Immunofluorescence analysis confirmed that neurons co-cultured with CIRP-treated microglial supernatant exhibited altered morphology and reduced axonal integrity, which was ameliorated upon TCC treatment (Fig. 6**F-G**).

### TCC reduces microglial activation and regulates polarization to inhibit inflammation *in vitro*

**Fig. 7:**
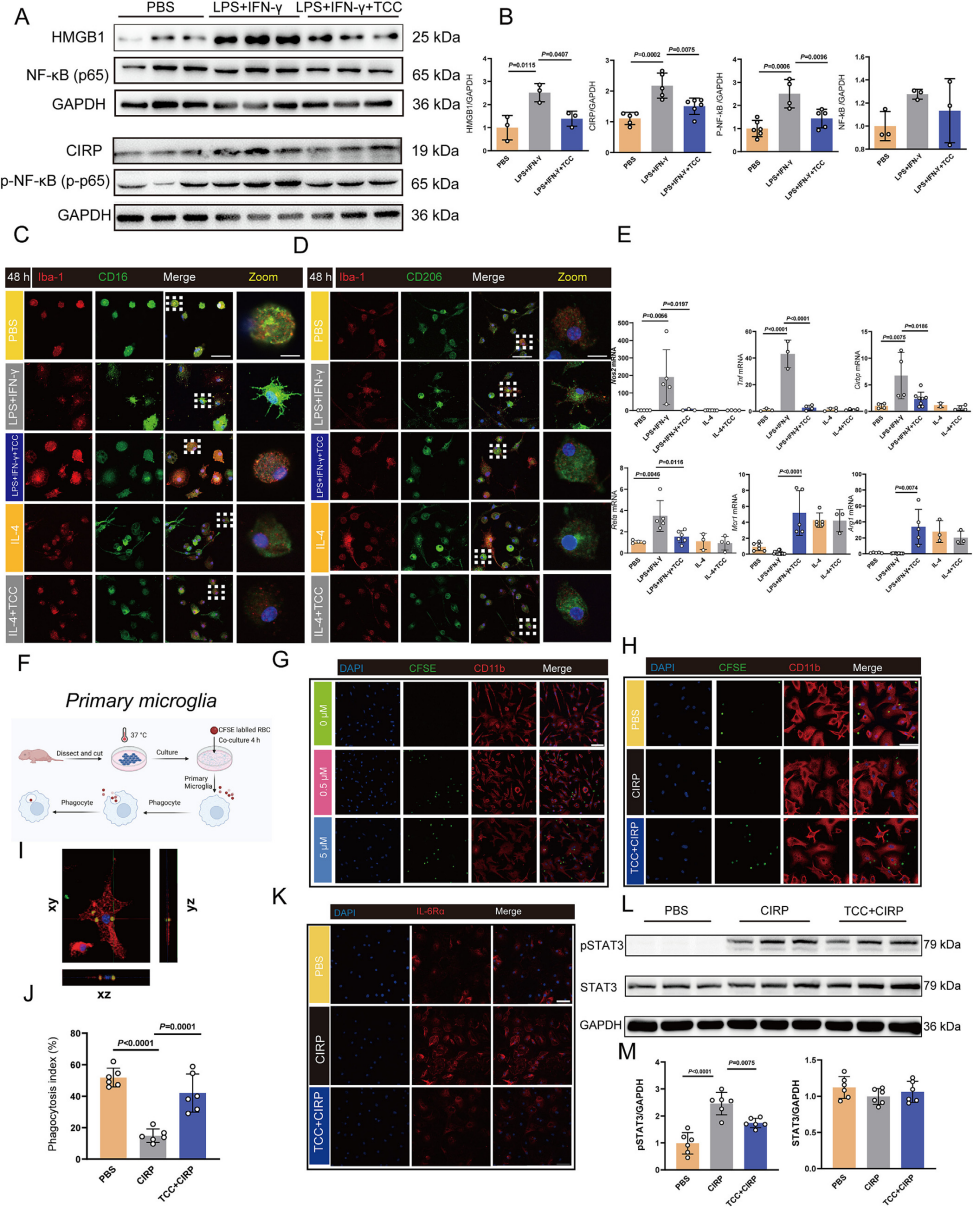
TCC reduces microglial activation and regulates polarization to inhibit inflammation, while TCC derived from CIRP enhances microglial phagocytosis of red blood cells *in vitro***A, B** Western blot analysis showing reduced release of HMGB1, p-NF-κB, CIRP, and NF-κB in primary microglia following inflammatory stimuli, with statistical charts. *n* = 3–6. **C, D** Immunofluorescence analysis of Iba-1^+^CD16^+^ and Iba-1^+^CD206^+^ microglia, showing morphological changes after TCC treatment; scale bar = 50 μm, zoom scale bar = 25 μm. **E** qRT-PCR analysis of TCC’s effect on *Nos2, Tnf, Rela, Mcr1, Arg1,* and *Cirbp* gene expression in microglia. *n* = 3–8. **F** Experimental setup: CFSE-labeled red blood cells co-cultured with microglia for 4 h. **G** Fluorescence images showing CFSE-labeled red blood cells (green), CD11b-labeled microglial membranes (red), and DAPI-labeled nuclei (blue) at different concentrations (0, 0.5, 5 μM) of CFSE-labeled red blood cells (RBCs). Scale bar = 50 μm. **H** Immunofluorescence images of microglial phagocytosis of RBCs following TCC intervention after CIRP stimulation. Scale bar = 50 μm. **I** Enlarged 3D image showing microglial phagocytosis of red blood cells in xz, yz, and xy planes. **J** Quantitative analysis of the microglial phagocytic index. *n* = 6. **K** Immunofluorescence images showing TCC's effect on IL-6Rα expression in microglia post-CIRP stimulation. Scale bar = 50 μm. **L** Western blot showing p-STAT3, STAT3, and β-actin protein bands after TCC treatment. **M** Quantitative analysis of p-STAT3/STAT3 levels. *n* = 6.

To directly observe the impact of TCC on microglial inflammatory responses, we conducted primary microglial cell culture experiments. In Fig. 7**A and B**, TCC suppressed the release of inflammatory cytokines, including HMGB1 and CIRP, from primary microglia stimulated by LPS and IFN-γ, and inhibited NF-κB nuclear translocation. Immunofluorescence analysis of primary microglia subtypes further demonstrated that TCC regulated microglial polarization. Microglia stimulated with LPS and IFN-γ exhibited small protrusions and high CD16 expression, indicating M1 polarization. After TCC treatment, these protrusions diminished, and microglial morphology resembled that of the PBS group. In contrast, microglia stimulated with IL-4, which promotes M2 polarization, maintained a morphology similar to that of the PBS group after TCC treatment (Fig. 7**C, D**). Gene expression analysis after 48 h of treatment revealed that LPS and IFN-γ stimulated microglia to highly express *Nos2*, *Tnf*, *Cirbp*, and *Rela*. Following TCC treatment, expression of M2 markers *Mcr1* and *Arg1* increased, while *Nos2*, *Tnf*, *Cirbp*, and *Rela* expression decreased (Fig. 7E).

### TCC inhibits the IL-6R/JAK/STAT3 pathway to exert neuroprotective effects

To elucidate the molecular mechanisms underlying TCC's regulation of inflammatory responses, we further employed single-cell sequencing selected microglia for further analysis. Among the pathways compared between the ICH and TCC groups, the top 10 significantly enriched pathways were identified through GO enrichment analysis (**Fig. S4**A). The results showed that TCC treatment altered the enrichment pattern of inflammation-related pathways in microglia. In **Fig. S4**A, after TCC treatment, KEGG analysis revealed that there was minimal or no enrichment in IL-6r-JAK-STAT pathway, suggesting that TCC inhibited the activation of the IL-6r-JAK-STAT pathway by rendering the differential genes insignificant.

To further explore the regulatory effects of TCC on the IL-6Rα/STAT3 signaling pathway post-hemorrhage, we examined protein expression in the striatum on the third day after ICH using Western blot. The results showed that CIRP protein levels in the striatum were elevated on day 3 post-hemorrhage, but TCC treatment significantly reduced CIRP expression (**Fig. S4**B). Additionally, both IL-6Rα and pSTAT3 protein levels increased post-hemorrhage, while TCC treatment led to a reduction in IL-6Rα expression and decreased STAT3 phosphorylation (**Fig. S4**B). These findings suggest that CIRP released in the perihematomal tissue activates the IL-6Rα/STAT3 pathway post-ICH, and TCC exerts its neuroprotective effects by downregulating this signaling pathway.

### TCC enhances microglial phagocytosis of RBCs by inhibiting CIRP-induced IL-6Rα/STAT3 pathway

The functional characteristics of microglial cells encompass not only the release of inflammatory cytokines but also their phagocytic activity. To investigate the phagocytic function of microglia towards RBCs *in vitro*, we incubated microglia with 5(6)-carboxyfluorescein diacetate (CFSE)-labeled erythrocytes at a ratio of 10:1 (erythrocytes to phagocytes) for 4 h (Fig. 7F). RBCs were labeled with either 0.5 μM or 5 μM CFSE, and immunofluorescence analysis determined that 5 μM CFSE was the optimal concentration for labeling (Fig. 7G). Following this, we pretreated microglia with TCC, stimulated them with CIRP to induce inflammation, and then co-incubated them with CFSE-labeled RBCs. Immunofluorescence analysis showed that the number of RBCs phagocytosed by microglia was significantly reduced in the CIRP group compared to the PBS group. However, pretreatment with TCC improved erythrocyte phagocytosis in the TCC + CIRP group (Fig. 7**H-J**). To further investigate whether TCC inhibits the binding of CIRP/IL-6Rα and the subsequent activation of the STAT3 signaling pathway, we examined IL-6Rα expression on microglia using immunofluorescence (Fig. 7K). In the PBS group, microglia showed almost no IL-6Rα expression. After CIRP stimulation, IL-6Rα expression on the microglial membrane significantly increased. However, in the TCC + CIRP group, IL-6Rα expression was notably reduced. Western blot analysis further demonstrated that CIRP stimulation led to an increase in the p-STAT3/GAPDH ratio compared to the PBS control group, while TCC treatment reduced p-STAT3 levels (Fig. 7**L, M**). These results indicate that TCC inhibits the upregulation of the IL-6Rα/STAT3 pathway in microglia following CIRP stimulation, thereby enhancing microglial phagocytosis of RBCs.

**Fig. 8:**
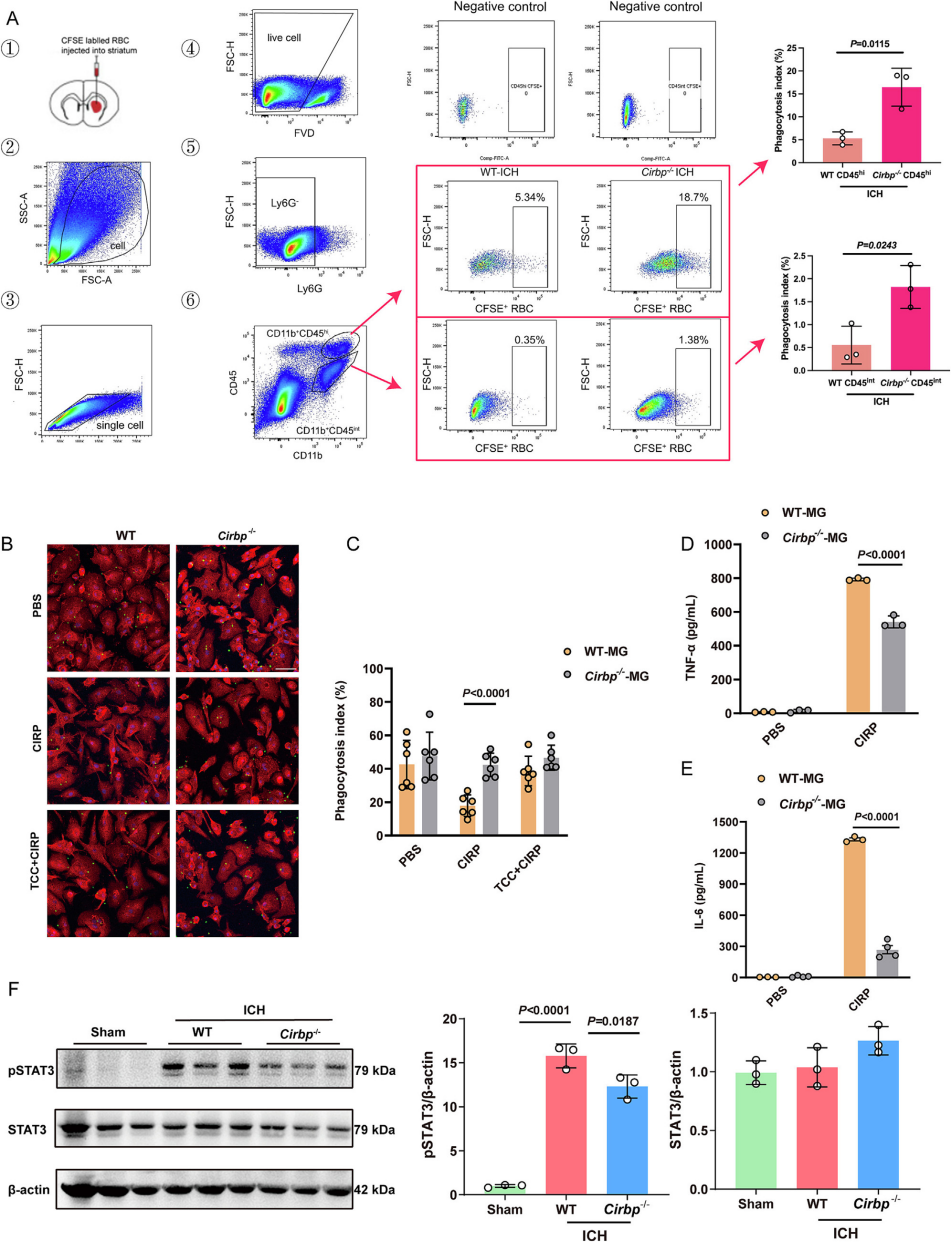
Knockout *Cirbp* attenuates microglial phagocytosis and pro-inflammatory cytokine levels via STAT3 pathway **A** CFSE-labeled RBCs were injected into the striatum (①), and cells were sequentially gated for total cells (②), singlets (③), live cells (④), Ly6G^−^ population (⑤), and CD11b^+^CD45^hi^ / CD11b^+^CD45^int^ subsets (⑥). Negative controls without RBC injection are shown to define CFSE baseline, and representative plots of WT-ICH and *Cirbp*^−^/^−^ ICH mice are presented with gating for CFSE^+^ events set beyond 10^4^. The bar graphs on the right summarize the phagocytosis index (%) of each group. *n* = 3. **B, C** Immunofluorescence images of WT and *Cirbp*^−/−^ microglia phagocytosing RBCs. Green: CFSE-labeled RBCs, Red: CD11b-labeled microglial membranes, Blue: DAPI-labeled nuclei. Scale bar = 50 μm. Quantitative analysis of microglial phagocytosis post-rmCIRP and TCC stimulation. **D, E** ELISA analysis of TNF-α and IL-6 levels in the culture supernatant of microglia from WT and *Cirbp*^−/−^ mice at 24 h. *n* = 3–4. **F** Western blot analysis of p-STAT3, STAT3, and β-actin expression in the striatum of WT and *Cirbp*^−/−^ mice on day 3 post-ICH. *n* = 3.

To further investigate whether *Cirbp*^−/−^ mice exhibit enhanced hematoma clearance and improved neurological recovery following ICH, we assessed erythrophagocytosis in the brain using flow cytometry. RBCs were isolated via density gradient centrifugation and labeled with CFSE before being stereotactically injected into the right striatum. Three days post-injection, mice were anesthetized and perfused transcardially with ice-cold PBS. Brain tissues were dissociated into single-cell suspensions and stained with antibodies against Ly6G, CD45, and CD11b. Cell viability was assessed using a Fixable Viability Dye, and flow cytometric analysis was subsequently performed. Flow cytometry identified two major CD45-expressing populations within the Ly6G^−^ myeloid compartment: CD45^int^CD11b^+^ resting microglia and CD45^hi^CD11b^+^ activated microglia/macrophages (Fig. 8A). Quantitative analysis revealed that the number of CFSE^+^Ly6G^−^CD45^hi^CD11b^+^ activated microglia/macrophages was significantly higher in *Cirbp*^−/−^ mice compared to WT controls (Fig. 8A). The number of CFSE^+^Ly6G^−^CD45^int^CD11b^+^ resting microglia was also elevated in *Cirbp*^−/−^ mice (Fig. 8A). Notably, in both genotypes, the number of CFSE^+^ activated microglia/macrophages far exceeded that of CFSE^+^ resting microglia, suggesting that activated microglia/macrophages are the predominant phagocytic cell type responsible for hematoma clearance following ICH. Further, we stimulated WT and *Cirbp*^−/−^ microglia with rmCIRP for 24 h, followed by incubation with CFSE-labeled erythrocytes. Immunofluorescence observations confirmed that, after 24 h of stimulation with rmCIRP, *Cirbp*^−/−^ microglia had a greater capacity to phagocytize CFSE-labeled erythrocytes compared to WT microglia (Fig. 8**B,C**). ELISA analysis showed TNF-α and IL-6 concentrations in microglia cultures derived from WT and *Cirbp*^−/−^ mice following a 24-hour period. Significantly, under CIRP treatment, *Cirbp*^−/−^ microglia demonstrated a marked decrease in the secretion of both IL-6 and TNF-α levels compared to their WT counterparts (Fig. 8**D,E**). In addition, pSTAT3/β-actin levels were significantly reduced in *Cirbp*^-/-^ mice compared to WT mice post-ICH (Fig. 8F).

### TCC inhibits microglial inflammatory response via STAT3 in an ICH mouse model

Three doses of collagenase IV (0.6 U/kg, 1.2 U/kg, and 2.4 U/kg) were tested to induce hematoma formation. The 0.6 U/kg group had the highest survival rate compared to the higher doses, with increased mortality observed at higher doses (**Fig. S5**A). Neurological assessments, including mNSS, rotarod, and grid walking tests, indicated that the 1.2 U/kg group experienced more severe neurological impairment than the 0.6 U/kg and sham groups in short terms during 7 days (**Fig. S5**B-D). To assess long-term sensorimotor changes after ICH, we measured mNSS scores and rotarod performance on days 14, 21, and 28 post-injury. Both 1.2 U/kg and 0.6 U/kg collagenase IV groups had higher mNSS scores than Sham (**Fig. S5**E). On the rotarod, 1.2 U/kg ICH mice had shorter retention times than Sham on all days tested (**Fig. S5**E). These findings indicate that 1.2 U/kg collagenase IV impairs long-term sensorimotor function. Hematoma volume was larger in the 1.2 U/kg group compared to the 0.6 U/kg group on days 3 and 7 (**Fig. S5**F-G). Immunofluorescence analysis revealed increased microglial activation in the striatum of ICH mice, particularly in the 1.2 U/kg group (**Fig. S5**H-I). Neuronal counts in the area surrounding the hematoma were significantly lower in the 1.2 U/kg group compared to the sham and 0.6 U/kg groups (**Fig. S5**H-I). Additionally, western blot analysis revealed decreased phosphorylated STAT3 (p-STAT3) expression, indicating that TCC suppresses the IL-6R/ STAT3 pathway (**Fig. S5**J).

### Genetic depletion of IL6Rα reduces pro-inflammatory cytokine levels, while concurrently improving sensory and behavioral deficits and reducing hematoma volume following ICH

**Fig. 9:**
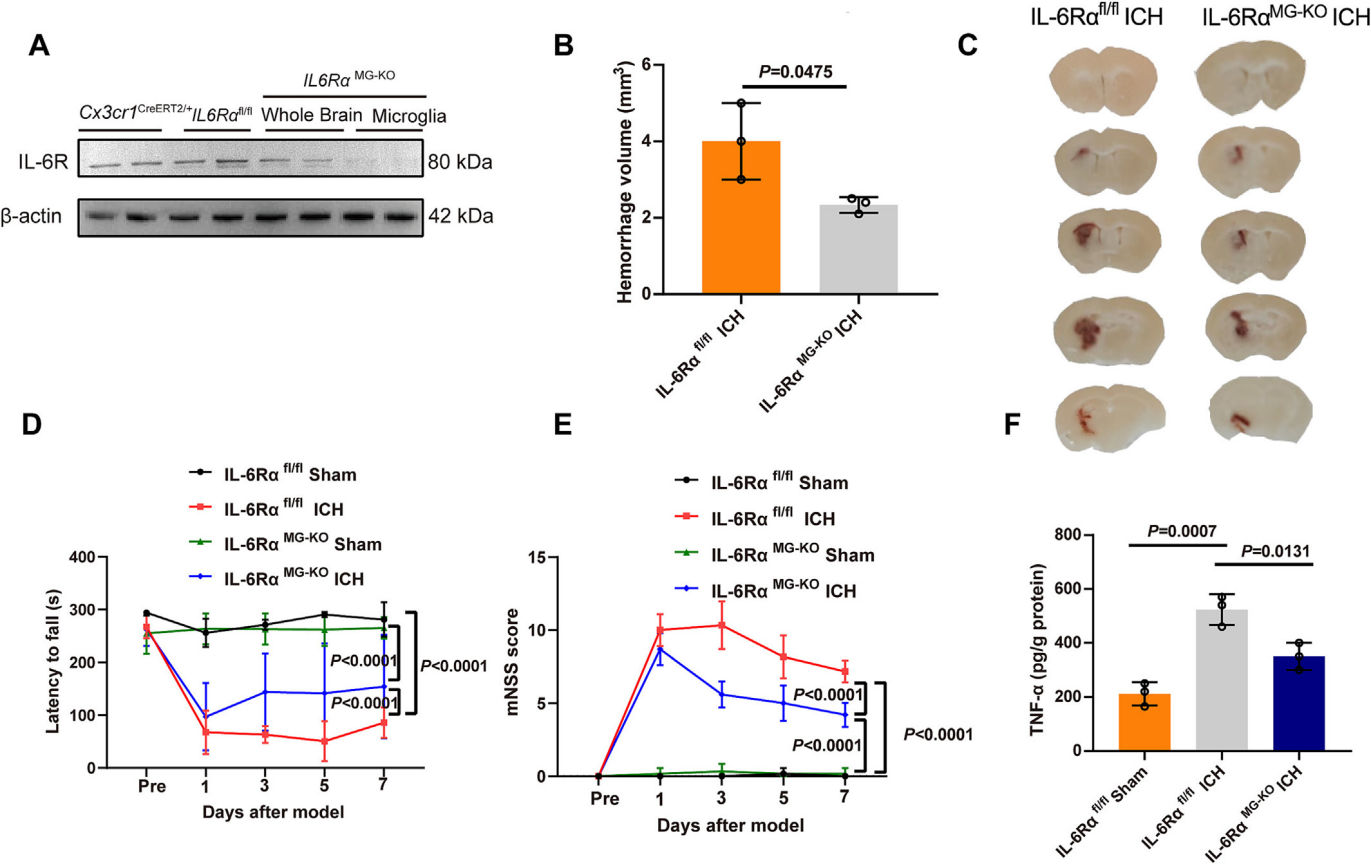
Microglia-specific genetic deletion of IL-6Rα reduces pro-inflammatory cytokine levels, while concurrently improving sensory and behavioral deficits and reducing hematoma volume following ICH **A** The expreesion of IL-6R in *Cx3cr1*^CreERT2/+^, *IL6Rα*^fl/fl^ and *IL6Rα*^MG-KO^ (whole brain tissue and microglia tissue respectively) mice was tested by western blot. **B, C** Coronal brain slices from *IL6Rα*^MG-KO^ mice showing hematoma levels post-ICH, with hemorrhage volume quantified. *n* = 3. **D,E** Neurological behavior tests (mNSS score, rotarod test) in microglia-specific *IL6Rα* knockout (*IL6Rα*^MG-KO^) mice after ICH, assessed on days 1,3,5,7. *n* = 3–6. **F** ELISA measurement of TNF-α levels in the striatum on day 7 post-ICH. *n* = 3.

We constructed an inducible conditional gene knockout mouse model. We established a collagenase IV-induced ICH model in adult male WT and *IL6Rα* microglia-specific knockout (*IL6Rα*^MG-KO^) mice. Firstly, we tested the expression of IL-6R in *Cx3cr1*^CreERT2/+^, *IL6Rα*^fl/fl^ and *IL6Rα*^MG-KO^ (whole brain tissue and microglia tissue respectively) mice by western blot (Fig. 9A). Coronal brain slices of *IL6Rα* ^MG-KO^ mice showed significantly smaller hematoma volumes compared to WT mice on day 7 post-ICH (Fig. 9**B,C**). Neurological behaviors, including mNSS scores and rotarod performance, were then assessed post-ICH (Fig. 9**D,E**). There were no differences between sham-operated WT and *IL6Rα*^MG-KO^ mice. However, after ICH, *IL6Rα*^MG-KO^ mice exhibited significantly lower mNSS scores compared to WT mice, indicating improved neurological outcomes. In the rotarod test, *IL6Rα*^MG-KO^ mice tended to stay on the rotarod longer post-ICH compared to WT mice, though this difference was not statistically significant. ELISA analysis of TNF-α levels in the striatum revealed higher TNF-α expression in the WT ICH group compared to the sham group, but no significant difference was observed between WT and *IL6Rα*^MG-KO^ mice post-ICH (Fig. 9F).

## Discussion

Our data indicated that differentially expressed genes in ICH were enriched in the IL-6/JAK/STAT pathway, a critical signaling cascade in inflammation. Previous studies have shown that MD2 binds to a specific 15-amino acid segment of CIRP with high affinity, promoting inflammation [26]. To target this interaction, we designed the peptides TCC and TC using macromolecular docking techniques, with SPR confirming that TCC reduced the binding affinity of CIRP to IL-6Rα. Overexpression studies showed that IL-6R exacerbates CIRP expression, and co-immunoprecipitation experiments demonstrated that TCC effectively inhibits CIRP-IL-6Rα binding, thereby reducing downstream STAT3 phosphorylation. This mechanism aligns with previous findings that IL-6Rα serves as a high-affinity receptor for CIRP in macrophages, and that targeting CIRP-IL-6Rα interactions could suppress neuroinflammation [34,39]. Similar results were observed with the peptide C23, which inhibits CIRP-induced activation of the IL-6Rα/STAT3/Cdk5 pathway in neuronal cells [40].

In our study, the Tat sequence in TCC facilitated its efficient crossing of the blood–brain barrier, allowing widespread distribution in the brain, particularly in neurons, as shown by fluorescein-tagged TCC imaging. This is critical for targeting regions of hemorrhage and injury where blood–brain barrier permeability is increased following ICH [41,42]. In contrast, the therapeutic efficacy of IL-6R–targeting antibodies in the central nervous system remains uncertain, as these antibodies, such as the Rat IgG2b anti-IL-6R (150 kDa), are typically unable to cross the BBB under normal physiological conditions. Large molecules like IgG rarely penetrate the BBB passively, unless facilitated by active transport mechanisms or pathological conditions that compromise barrier integrity. In our study, the TCC peptide demonstrated the capacity to effectively traverse the BBB and antagonize IL-6R within the brain, offering a potential advantage over conventional antibody-based therapies for modulating neuroinflammation following ICH. Our data showed that TCC reduced CIRP, IL-6R expression, attenuating the excessive inflammatory response in brain tissue, and enhancing axonal regeneration, neuronal repair, and white and gray matter recovery. Furthermore, TCC treatment significantly improved both short- and long-term motor function recovery and reversed cognitive dysfunction in the Morris water maze test. These results align with previous research demonstrating the neuroprotective effects of CIRP inhibition in ischemic stroke and traumatic brain injury models [25]. Our study also explored the molecular mechanisms underlying TCC's neuroprotective effects, focusing on the inflammatory profile of microglia. TCC treatment markedly suppressed post-hemorrhagic microglial activation, reducing the release of inflammatory mediators such as HMGB1, CIRP, and NF-κB. It has been shown that ethanol exposure activates microglia to produce CIRP, leading to a pro-inflammatory response and exacerbating neuroinflammation[[43], [44], [45]].

Microglial polarization plays a pivotal role in the progression and resolution of neuroinflammation[46,47]. While M1 microglia promote inflammation and tissue damage, M2 microglia support tissue repair and anti-inflammatory responses [16,20,23]. In ICH models, M1 cytokines such as TNF-α and IL-1β rise within the first day, driven by NF-κB activation[48,49], while anti-inflammatory cytokines like IL-4 and TGF-β1 support M2 polarization and functional recovery[[50], [51], [52]]. However, the specific role of CIRP in regulating microglial polarization has been underexplored[53]. In our study, TCC treatment shifted microglial polarization toward an anti-inflammatory phenotype, as evidenced by increased CD206 expression and reduced CD16 expression, along with decreased TNF-α levels. This shift likely contributes to the therapeutic effects of TCC by promoting M2-like microglia, which facilitate tissue repair. Additionally, *Cirbp* knockout mice exhibited enhanced microglial phagocytosis and reduced inflammatory responses, further supporting the role of CIRP in negatively regulating microglial function during brain hemorrhage. Single-cell sequencing and pathway enrichment analyses confirmed that TCC treatment downregulated inflammation-related signaling pathways, particularly the IL-6R/JAK/STAT3 pathway, which has been implicated in neuroinflammation and is a promising therapeutic target. Previous studies have shown that extracellular CIRP activates the neurotoxic cyclin-dependent kinase-5 (Cdk5)/p25 pathway through the IL-6Rα/STAT3 pathway, linking CIRP to neurodegenerative diseases such as Alzheimer's disease[40,54]. In our model, targeting the CIRP-IL-6R interaction effectively suppressed this pathway, offering neuroprotection following ICH.

The absence of endogenous CIRP appears to fundamentally alter the inflammatory response of microglia to exogenous CIRP stimulation. Immunofluorescence analysis revealed that *Cirbp*^−/−^ microglia exhibited a significantly enhanced capacity for phagocytosis of CFSE-labeled erythrocytes compared to WT microglia following 24 h of rmCIRP stimulation. This suggests that the lack of endogenous CIRP may prime microglia for more robust phagocytic activity, potentially as a compensatory mechanism. Moreover, the ELISA results further demonstrated that *Cirbp*^−/−^ microglia secreted significantly lower levels of pro-inflammatory cytokines, IL-6 and TNF-α, compared to WT microglia when treated with exogenous CIRP. This indicates that endogenous CIRP is likely a key driver of inflammation in microglia, and its absence may dampen the cellular pathways responsible for cytokine production in response to external CIRP stimuli. These findings suggest that CIRP's role in the inflammatory cascade is multifaceted, acting both as a mediator of inflammation and as a regulator of microglial functional responses. The reduced cytokine secretion and enhanced phagocytosis in *Cirbp*^−/−^ microglia might reflect a shift in microglial activation states, favoring resolution of inflammation over propagation. This highlights the potential for therapeutic modulation of CIRP signaling to attenuate excessive inflammatory responses in pathological conditions.

Apart from that, this study demonstrates that CIRP, along with IL-6, and TNF-α, are significantly elevated in the peripheral blood of patients with ICH. The elevated levels of these inflammatory markers, especially CIRP, suggest a robust inflammatory response following hemorrhagic stroke. CIRP, which is normally expressed at low levels in the brain, becomes significantly upregulated during stroke and promotes inflammation through the TLR4 pathway[25,26]. The increase in NIHSS score in ICH patients aligns with the elevated levels of inflammatory markers, supporting the hypothesis that inflammation contributes to worse neurological outcomes. Elevated IL-6 and TNF-α levels, known to be involved in neuroinflammatory responses, might further drive neuronal damage, while MD2 may facilitate TLR4-mediated inflammatory pathways. As an extracellular DAMP, CIRP forms a complex with MD2 and TLR4, activating NF-κB signaling and upregulating pro-inflammatory cytokines such as TNF-α and HMGB1 [29,45,55,56]. Previous studies have shown that CIRP upregulation in stroke models correlates with increased TNF-α expression and brain injury [55]. This response may contribute to secondary brain injury by exacerbating edema and increasing intracranial pressure, as observed in the CT imaging. Together, these findings highlight the role of inflammation in ICH pathophysiology and suggest that CIRP and related inflammatory markers could serve as targets for therapeutic intervention to mitigate secondary brain injury. Our findings align with previous research, as we successfully established a stable ICH model by injecting collagenase IV into the striatum, which allowed us to investigate the molecular mechanisms underlying ICH-induced neuroinflammation[20]. Sequencing data and immunofluorescence analysis demonstrated high CIRP expression in immune cells, particularly neurons, following ICH. This evidence supports the hypothesis that CIRP contributes to neuronal injury by promoting inflammatory responses. Despite its significant findings, this study has several limitations. While the neuroprotective effects of TCC were demonstrated in preclinical models, its pharmacokinetics, long-term safety, and translational applicability require further investigation. Additionally, rodent models were used, which may not completely replicate the heterogeneity and immune responses of human ICH. The clinical validation of CIRP’s role was correlational, lacking causative evidence linking CIRP levels to patient outcomes. Furthermore, the effects of CIRP and TCC on other CNS cell types, such as astrocytes and neurons, remain incompletely understood. Addressing these limitations in future research will be critical to advancing the therapeutic potential of CIRP-targeted interventions for ICH.

## Conclusion

This study provides strong evidence that targeting CIRP and IL-6R can significantly attenuate neuroinflammation and enhance recovery following ICH. Our findings demonstrate that the CIRP-targeting peptide Tat-CIRP CMA (TCC) effectively reduces inflammatory responses by inhibiting the IL-6R/JAK/STAT3 pathway, modulating microglial polarization, and enhancing neuronal protection. Knockout *Cirbp* attenuates microglial phagocytosis and pro-inflammatory cytokine levels via STAT3 pathway. Furthermore, genetic depletion of *IL-6Rα* in microglia reduces pro-inflammatory cytokine levels, while concurrently improving sensory and behavioral deficits and reducing hematoma volume following ICH, underscoring the critical role of CIRP and IL-6R in exacerbating neuroinflammation. TCC treatment not only improved sensorimotor and cognitive function but also preserved white and gray matter integrity, suggesting its potential as a therapeutic strategy for ICH.

## Ethical approval

This study was performed in line with the principles of the Declaration of Helsinki. Approval was granted by the Animal Ethics Committee of Nantong University and Medical Ethics Committee of Henan Provincial People's Hospital and Affiliated Hospital of Nantong University.

## Consent to participate

Informed consent was obtained from all individual participants included in the study.

## Data availability

The scRNA-seq data underpinning the discoveries of this study are archived in the Sequence Read Archive (SRA) database under the accession code PRJNA1005089. Other data that support the findings of this study are available from the corresponding author upon reasonable request.

## Compliance with Ethics Requirements

All Institutional and National Guidelines for the care and use of animals (fisheries) were followed.

## Funding

This work was supported by the National Natural Science Foundation of China (Grants 82171190 and 81873924), Major National Science and Technology Special Project of China (Grant 2023ZD0505304), Henan Center for Outstanding Overseas Scientists (Grant number: GZS2022019), Henan Province science and technology research project (Grant number: 242102311095), and China Postdoctoral Science Foundation (2020M673649).

## Declaration of competing interest

The authors declare that they have no known competing financial interests or personal relationships that could have appeared to influence the work reported in this paper.

## References

[1] Zhu H., Wang Z., Yu J., Yang X., He F., Liu Z. (2019). Role and mechanisms of cytokines in the secondary brain injury after intracerebral hemorrhage. Prog Neurobiol.

[2] Reyes R., Viswanathan M., Aiyagari V. (2019). An update on neurocritical care for intracerebral hemorrhage. Expert Rev Neurother.

[3] Meretoja A., Strbian D., Putaala J., Curtze S., Haapaniemi E., Mustanoja S. (2012). SMASH-U: a proposal for etiologic classification of intracerebral hemorrhage. Stroke.

[4] Shao A., Zhu Z., Li L., Zhang S., Zhang J. (2019). Emerging therapeutic targets associated with the immune system in patients with intracerebral haemorrhage (ICH): from mechanisms to translation. EBioMedicine.

[5] Lord A.S., Gilmore E., Choi H.A., Mayer S.A. (2015). Time course and predictors of neurological deterioration after intracerebral hemorrhage. Stroke.

[6] Babu R., Bagley J.H., Di C., Friedman A.H., Adamson C. (2012). Thrombin and hemin as central factors in the mechanisms of intracerebral hemorrhage-induced secondary brain injury and as potential targets for intervention. Neurosurg Focus.

[7] Zhou Y., Wang Y., Wang J., Anne Stetler R., Yang Q.W. (2014). Inflammation in intracerebral hemorrhage: from mechanisms to clinical translation. Prog Neurobiol.

[8] Zhang Z., Zhang Z., Lu H., Yang Q., Wu H., Wang J. (2017). Microglial Polarization and Inflammatory Mediators after Intracerebral Hemorrhage. Mol Neurobiol.

[9] Nayak D., Roth T.L., McGavern D.B. (2014). Microglia development and function. Annu Rev Immunol.

[10] Ginhoux F., Greter M., Leboeuf M., Nandi S., See P., Gokhan S. (2010). Fate mapping analysis reveals that adult microglia derive from primitive macrophages. Science (New York, NY).

[11] Azam S., Haque M.E., Kim I.S., Choi D.K. (2021). Microglial turnover in Ageing-Related Neurodegeneration: Therapeutic Avenue to Intervene in Disease Progression. Cells.

[12] Subhramanyam C.S., Wang C., Hu Q., Dheen S.T. (2019). Microglia-mediated neuroinflammation in neurodegenerative diseases. Semin Cell Dev Biol.

[13] Hanisch U.K., Kettenmann H. (2007). Microglia: active sensor and versatile effector cells in the normal and pathologic brain. Nat Neurosci.

[14] Cherry J.D., Olschowka J.A., O'Banion M.K. (2014). Neuroinflammation and M2 microglia: the good, the bad, and the inflamed. J Neuroinflammation.

[15] Wang G., Zhang J., Hu X., Zhang L., Mao L., Jiang X. (2013). Microglia/macrophage polarization dynamics in white matter after traumatic brain injury. J Cereb Blood Flow Metab.

[16] Hu X., Leak R., Shi Y., Suenaga J., Gao Y., Zheng P. (2015). Microglial and macrophage polarization—new prospects for brain repair. Nat Rev Neurol.

[17] Morgan S.C., Taylor D.L., Pocock J.M. (2004). Microglia release activators of neuronal proliferation mediated by activation of mitogen-activated protein kinase, phosphatidylinositol-3-kinase/Akt and delta-Notch signalling cascades. J Neurochem.

[18] Liao H., Bu W.Y., Wang T.H., Ahmed S., Xiao Z.C. (2005). Tenascin-R plays a role in neuroprotection via its distinct domains that coordinate to modulate the microglia function. J Biol Chem.

[19] Hu X., Li P., Guo Y., Wang H., Leak R., Chen S. (2012). Microglia/macrophage polarization dynamics reveal novel mechanism of injury expansion after focal cerebral ischemia. Stroke.

[20] Ye L., Tang X., Zhong J., Li W., Xu T., Xiang C. (2024). Unraveling the complex pathophysiology of white matter hemorrhage in intracerebral stroke: A single-cell RNA sequencing approach. CNS Neurosci Ther.

[21] Liu J., Peng S., Ye L., Sun Y., Zhao Q., Wei H. (2023). Neuroinflammation aggravated by traumatic brain injury at high altitude is reversed by L-serine via NFAT1-mediated microglial polarization. Front Cell Neurosci.

[22] Chio C.C., Lin M.T., Chang C.P. (2015). Microglial activation as a compelling target for treating acute traumatic brain injury. Curr Med Chem.

[23] Wang G., Shi Y., Jiang X., Leak R., Hu X., Wu Y. (2015). HDAC inhibition prevents white matter injury by modulating microglia/macrophage polarization through the GSK3β/PTEN/Akt axis. Proc Natl Acad Sci U S A.

[24] Zhou K., Cui S., Duan W., Zhang J., Huang J., Wang L. (2020). Cold-inducible RNA-binding protein contributes to intracerebral hemorrhage-induced brain injury via TLR4 signaling. Brain Behav.

[25] Fang Z., Wu D., Deng J., Yang Q., Zhang X., Chen J. (2021). An MD2-perturbing peptide has therapeutic effects in rodent and rhesus monkey models of stroke. Sci Transl Med.

[26] Qiang X., Yang W., Wu R., Zhou M., Jacob A., Dong W. (2013). Cold-inducible RNA-binding protein (CIRP) triggers inflammatory responses in hemorrhagic shock and sepsis. Nat Med.

[27] Royster W., Jin H., Wang P., Aziz M. (2021). Extracellular CIRP decreases Siglec-G expression on B-1a cells skewing them towards a pro-inflammatory phenotype in sepsis. Molecular medicine (Cambridge, Mass).

[28] Li M., Yao M., Shao K., Shen X., Ge Z., Li Y. (2023). Serum cold-inducible RNA-binding protein (CIRP) levels as a prognostic indicator in patients with acute ischemic stroke. Front Neurol.

[29] Bolognese A.C., Sharma A., Yang W.L., Nicastro J., Coppa G.F., Wang P. (2018). Cold-inducible RNA-binding protein activates splenic T cells during sepsis in a TLR4-dependent manner. Cell Mol Immunol.

[30] Shen S.Y., Liang L.F., Shi T.L., Shen Z.Q., Yin S.Y., Zhang J.R. (2025). Microglia-Derived Interleukin-6 Triggers Astrocyte Apoptosis in the Hippocampus and Mediates Depression-like Behavior. Adv Sci (Weinh).

[31] Blecharz-Lang K.G., Wagner J., Fries A., Nieminen-Kelhä M., Rösner J., Schneider U.C. (2018). Interleukin 6-Mediated Endothelial Barrier Disturbances can Be Attenuated by Blockade of the IL6 Receptor Expressed in Brain Microvascular Endothelial Cells. Transl Stroke Res.

[32] Osuka K., Watanabe Y., Usuda N., Aoyama M., Iwami K., Takeuchi M. (2017). Inhibitory Mechanism of the Outer Membrane growth of Chronic Subdural Hematomas. J Neurotrauma.

[33] Ulfig N., Friese K. (1999). Interleukin-6 receptor is highly expressed in the ganglionic eminence of the human fetal brain. Biol Neonate.

[34] Zhou M., Aziz M., Denning N.L., Yen H.T., Ma G., Wang P. (2020). Extracellular CIRP induces macrophage endotoxin tolerance through IL-6R-mediated STAT3 activation. JCI Insight.

[35] Jiang H., Lu C., Wu H., Ding J., Li J., Ding J. (2024). Decreased cold-inducible RNA-binding protein (CIRP) binding to GluRl on neuronal membranes mediates memory impairment resulting from prolonged hypobaric hypoxia exposure. CNS Neurosci Ther.

[36] Jiang B.C., Ding T.Y., Guo C.Y., Bai X.H., Cao D.L., Wu X.B. (2022). NFAT1 Orchestrates Spinal Microglial Transcription and Promotes Microglial Proliferation via c-MYC contributing to Nerve Injury-Induced Neuropathic Pain. Adv Sci (Weinh).

[37] Ashburner M., Ball C.A., Blake J.A., Botstein D., Butler H., Cherry J.M. (2000). Gene ontology: tool for the unification of biology. The Gene Ontology Consortium Nature genetics.

[38] Kanehisa M., Goto S. (2000). KEGG: kyoto encyclopedia of genes and genomes. Nucleic Acids Res.

[39] Akama Y., Murao A., Aziz M., Wang P. (2024). Extracellular CIRP induces CD4CD8αα intraepithelial lymphocyte cytotoxicity in sepsis. Molecular medicine (Cambridge, Mass).

[40] Sharma A., Brenner M., Jacob A., Marambaud P., Wang P. (2021). Extracellular CIRP Activates the IL-6Rα/STAT3/Cdk5 Pathway in Neurons. Mol Neurobiol.

[41] Okada T., Suzuki H., Travis Z.D., Zhang J.H. (2020). The Stroke-Induced Blood-Brain Barrier Disruption: Current Progress of Inspection Technique, Mechanism, and Therapeutic Target. Curr Neuropharmacol.

[42] Niazi S.K. (2023). Non-invasive drug delivery across the blood-brain barrier: a prospective analysis. Pharmaceutics.

[43] Rajayer S.R., Jacob A., Yang W.L., Zhou M., Chaung W., Wang P. (2013). Cold-inducible RNA-binding protein is an important mediator of alcohol-induced brain inflammation. PLoS One.

[44] Han J., Zhang Y., Ge P., Dakal T.C., Wen H., Tang S. (2023). Exosome-derived CIRP: an amplifier of inflammatory diseases. Front Immunol.

[45] Aziz M., Brenner M., Wang P. (2019). Extracellular CIRP (eCIRP) and inflammation. J Leukoc Biol.

[46] Lyu J., Xie D., Bhatia T.N., Leak R.K., Hu X., Jiang X. (2021). Microglial/Macrophage polarization and function in brain injury and repair after stroke. CNS Neurosci Ther.

[47] Xiong X.Y., Liu L., Yang Q.W. (2016). Functions and mechanisms of microglia/macrophages in neuroinflammation and neurogenesis after stroke. Prog Neurobiol.

[48] Zhang Z., Liu Y., Huang Q., Su Y., Zhang Y., Wang G. (2014). NF-κB activation and cell death after intracerebral hemorrhage in patients. Neurological sciences : official journal of the Italian Neurological Society and of the Italian Society of Clinical Neurophysiology.

[49] Wu C.H., Shyue S.K., Hung T.H., Wen S., Lin C.C., Chang C.F. (2017). Genetic deletion or pharmacological inhibition of soluble epoxide hydrolase reduces brain damage and attenuates neuroinflammation after intracerebral hemorrhage. J Neuroinflammation.

[50] Yang J., Ding S., Huang W., Hu J., Huang S., Zhang Y. (2016). Interleukin-4 Ameliorates the Functional Recovery of Intracerebral Hemorrhage through the Alternative Activation of Microglia/Macrophage. Front Neurosci.

[51] Taylor R.A., Chang C.F., Goods B.A., Hammond M.D., Mac Grory B., Ai Y. (2017). TGF-β1 modulates microglial phenotype and promotes recovery after intracerebral hemorrhage. J Clin Invest.

[52] Pérez S, Rius-Pérez S. Macrophage Polarization and Reprogramming in Acute Inflammation: A Redox Perspective. Antioxidants (Basel, Switzerland). 2022;11(7). 10.3390/antiox11071394 PMC9311967 35883885

[53] Xue Y., Nie D., Wang L.J., Qiu H.C., Ma L., Dong M.X. (2021). Microglial Polarization: Novel Therapeutic Strategy against Ischemic Stroke. Aging Dis.

[54] Sharma A., Brenner M., Wang P. (2020). Potential Role of Extracellular CIRP in Alcohol-Induced Alzheimer's Disease. Mol Neurobiol.

[55] Zhou M., Yang W.L., Ji Y., Qiang X., Wang P. (2014). Cold-inducible RNA-binding protein mediates neuroinflammation in cerebral ischemia. BBA.

[56] Godwin A., Yang W., Sharma A., Khader A., Wang Z., Zhang F. (2015). Blocking cold-inducible RNA-binding protein protects liver from ischemia-reperfusion injury. Shock (Augusta, Ga).

