# Supplementary material for: Targeting CIRP and IL-6R-mediated microglial inflammation to improve outcomes in intracerebral hemorrhage
Source: J Adv Res. 2025 Sep 9;84:235–55. doi: 10.1016/j.jare.2025.09.012 (PMC13227277; doi:10.1016/j.jare.2025.09.012)
Supplement: Supplementary Data 1 [file mmc1.docx]

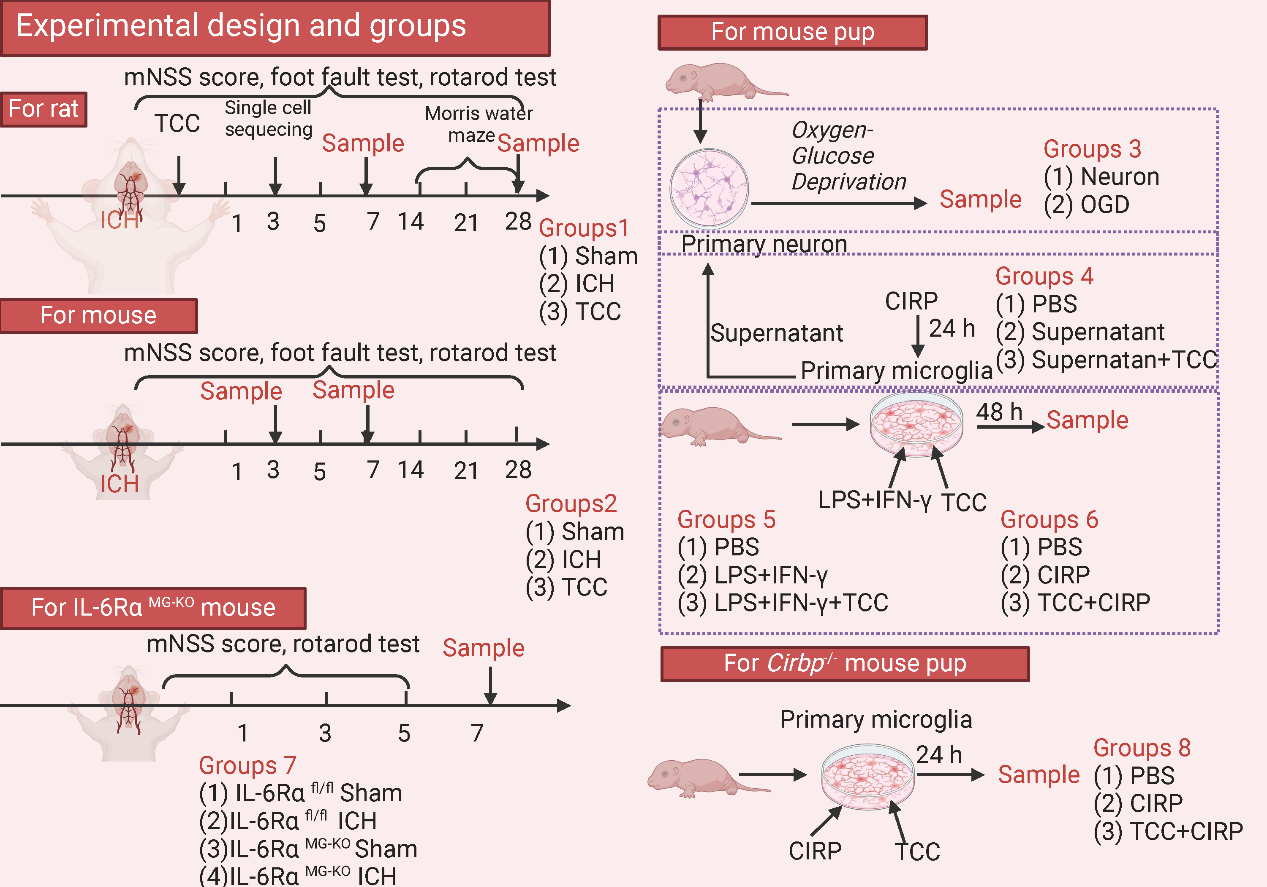


**Figure S1.** Schematic outline for the experimental design and groups *in vivo* and *in vitro*. ICH: Intracerebral Hemorrhage; mNSS: Modified Neurological Severity Score; TCC: Tat-CIRP-CMA; LPS: Lipopolysaccharide; IFN-γ: Interferon Gamma; PBS: Phosphate Buffered Saline; CIRP: Cold-Inducible RNA-Binding Protein.


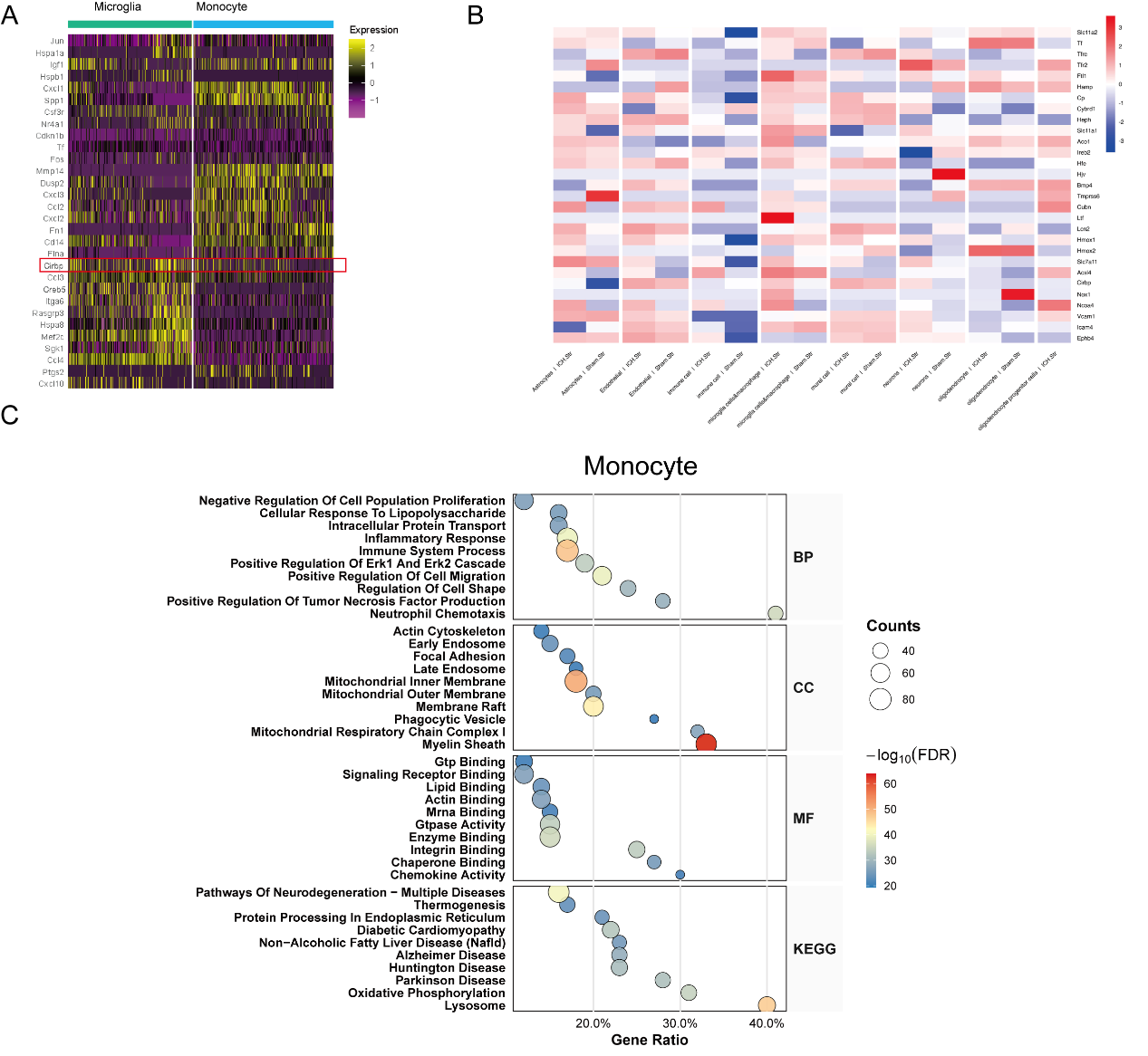


**Figure S2.** Single-cell sequencing analyses reveal inflammation-associated transcriptional changes in immune cells (microglia and monocyte) after ICH. **A** Heatmap illustrating changes in *Cirbp* and inflammatory genes expression of microglia and monocytes. **B** Heatmap illustrating changes in *Cirbp* and other genes expression in all type cells of brain in sham and ICH groups. **C** Gene Ontology (GO) enrichment analysis of differentially expressed genes (DEGs) in microglia, highlighting the top 10 significantly enriched biological processes, molecular functions and cellular component. and Kyoto Encyclopedia of Genes and Genomes (KEGG) pathway analysis of key pathways in monocytes from sham and ICH groups. Bubble plots are used to visualize the results of GO and KEGG enrichment analyses, respectively. The y-axis displays the top 10 significantly enriched terms or pathways, while the x-axis represents the gene ratio (the proportion of differentially expressed genes involved in each term). The size of each bubble corresponds to the number of enriched genes, and the color gradient reflects the statistical significance (adjusted p-value), with redder colors indicating higher significance.


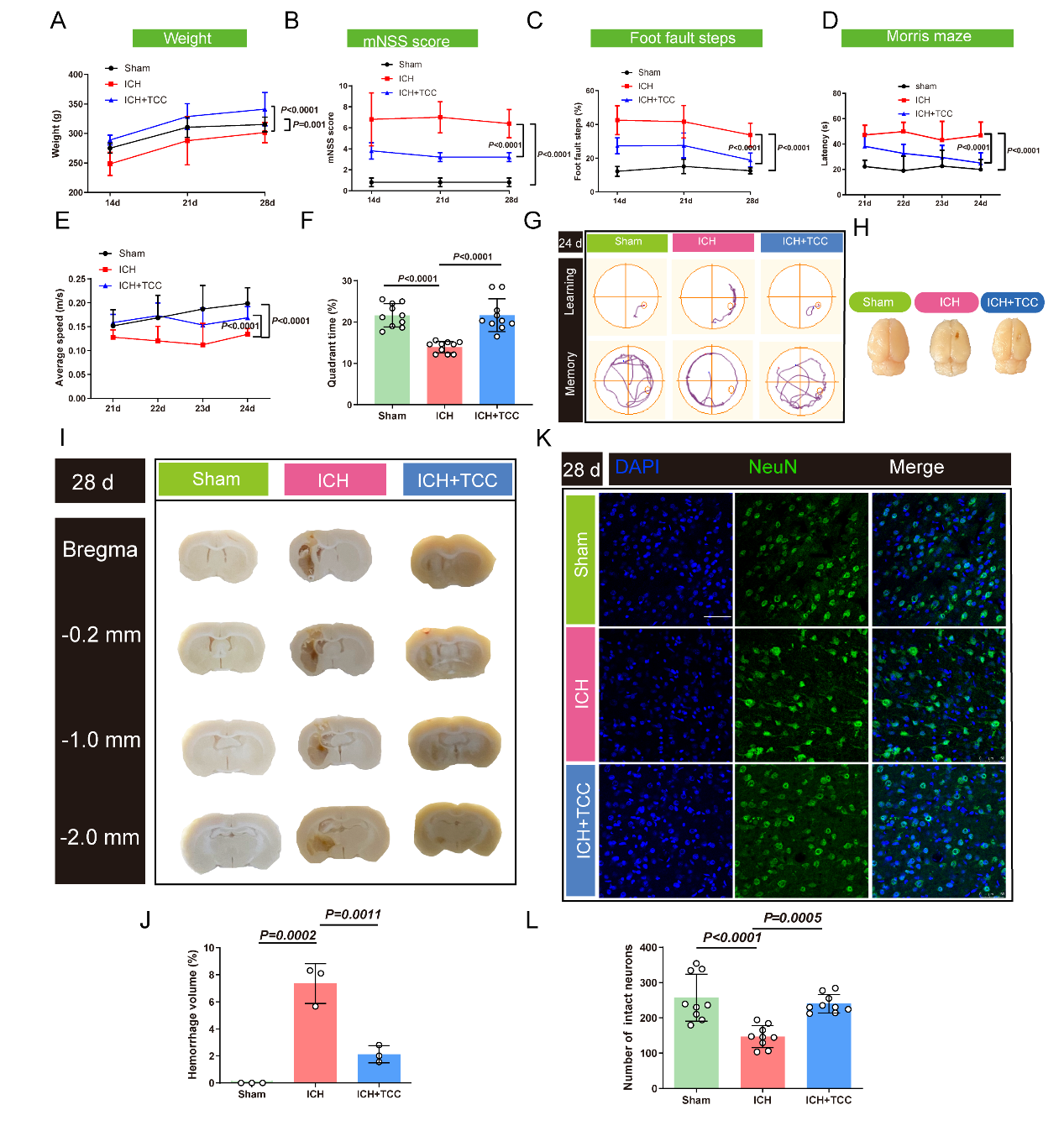


**Figure S3.** TCC provides long-term neuroprotection in ICH rats, enhancing sensorimotor function, reducing hemorrhage volume, and decreasing neuronal death. **A-C** TCC improves long-term sensorimotor function, as indicated by body weight and neurological behavior tests (mNSS score, foot fault test, corner turn test) at 14, 21, and 28 days post-ICH. *n* = 10. **D-G** Morris water maze results showing TCC’s effect on time to reach the target quadrant, average search speed, percentage of time spent in the target quadrant, and search trajectories post-hemorrhage. *n* = 4-6. **H-J** TCC reduces hematoma volume in the striatal region 28 days post-hemorrhage. *n* = 3. **K, L** NeuN immunofluorescence showing changes in cortical neurons on the hemorrhagic side 28 days post-TCC treatment, with intact neuron counts quantified; scale bar = 50 μm. *n* = 9.


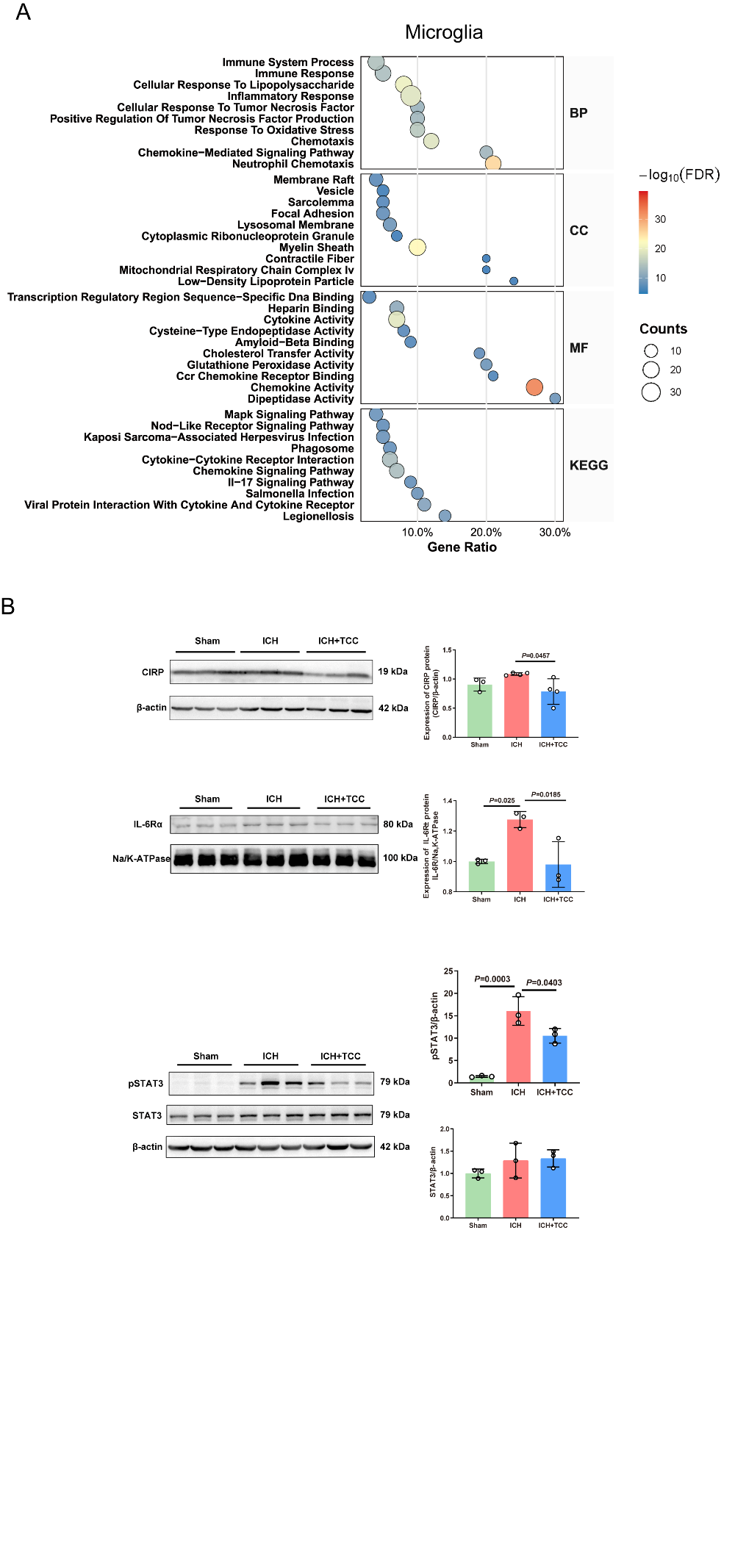


**Figure S4.** TCC inhibits the IL-6R/JAK/STAT3 pathway in microglia, providing neuroprotection. **A** GO enrichment analysis highlighting the top 10 significantly enriched pathways in microglia and KEGG pathway analysis of key pathways microglia from ICH and TCC groups. **B** Western blot analysis of ICH rats showing CIRP, β-actin, IL-6Rα, Na/K-ATPase, p-STAT3, and STAT3 protein bands in the striatum protein levels. *n* = 3-4.


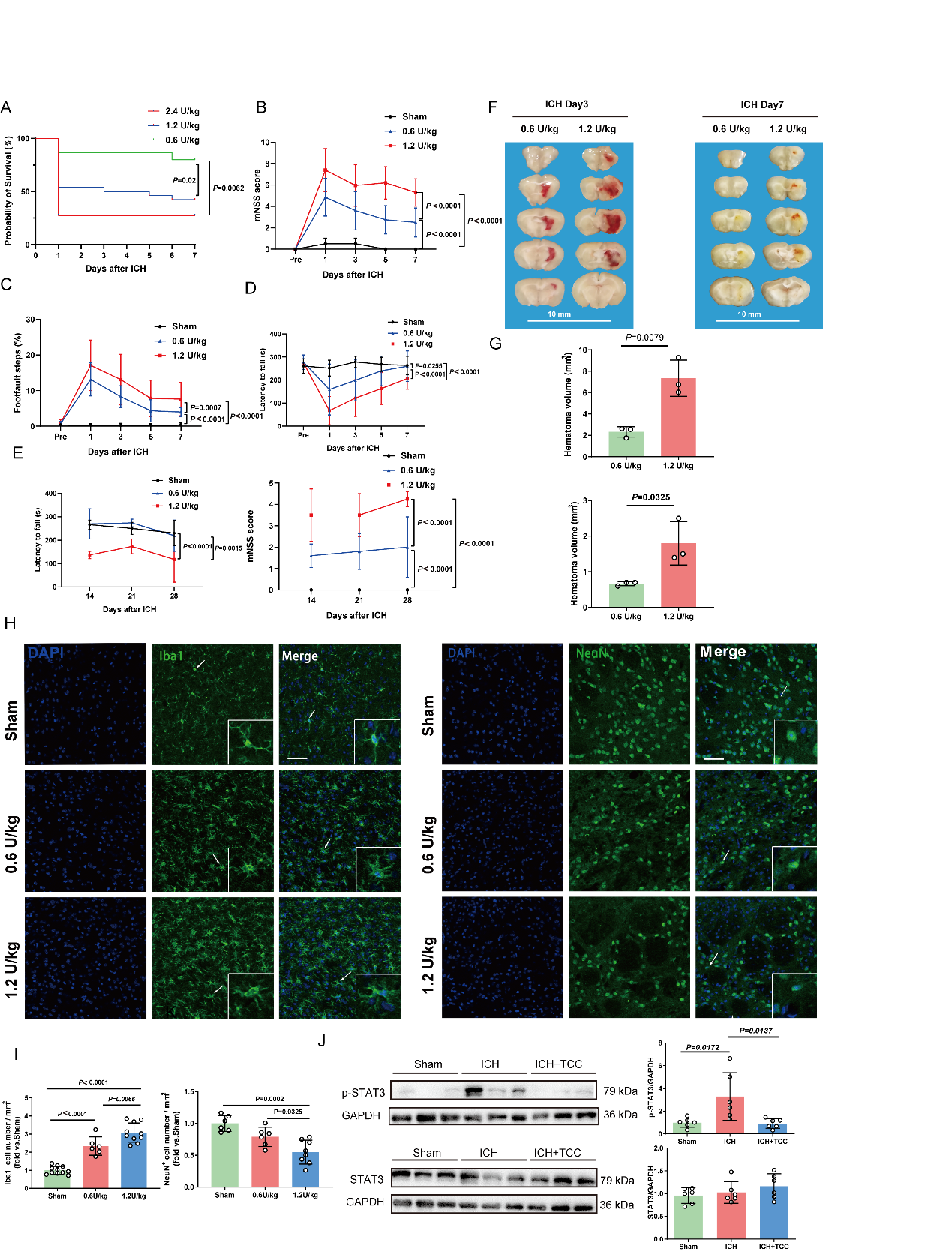


**Figure S5.** TCC suppresses microglial inflammatory response via STAT3 in ICH WT mice.

**A** Survival curve analysis of ICH mice induced by varying doses of collagenase IV over 7 days. The X-axis shows time points, and the Y-axis represents survival rate. *n* = 10. **B** Modified Neurological Severity Score (mNSS) on days 1, 3, 5, and 7 post-ICH. Y-axis indicates mNSS scores. *n* = 10. **C** Grid walking test results on days 1, 3, 5, and 7 post-ICH, with Y-axis representing the misstep rate of the left forelimb (%). *n* = 10. **D** Rotarod test results on days 1, 3, 5, and 7 post-ICH. Y-axis indicates time spent on the rotarod. *n* = 10. **E** Rotarod test results and mNSS scores on days 14, 21, and 28 post-ICH. Y-axis shows rotarod time. *n* = 4-7. **F** Representative coronal brain sections showing hematoma volume on days 3 and 7 post-ICH. Red indicates the hematoma; scale bar = 10 mm. **G** Quantification of hematoma volume on days 3 and 7 post-ICH. *n* = 3. **H-I** Immunofluorescence staining of striatum on day 7 post-ICH. Iba1 (green), NeuN (green), DAPI (blue); scale bar = 50 μm. Quantification of microglia/macrophages and neurons in the striatum on day 7 post-ICH. *n* = 6-11. **J** Quantitative analysis of pSTAT3 and STAT3 protein levels. *n* = 6.

**Table. S1 Basic Clinical Information of Patients with Intracerebral Hemorrhage (ICH)**

| Patient Number | Gender | Age | Comorbidities | Hemorrhage Location | Collection  of blood  after ICH (hours) | CT Scan Time  (hours) | Hemorrhage Volume (ml) |
| --- | --- | --- | --- | --- | --- | --- | --- |
| 1 | Male | 55 | Fatty Liver | Right Lateral Ventricle | 3 | 14 | 10 |
| 2 | Male | 36 | Hypertension | Left Parieto-Occipital Lobe | 4 | 5 | 35 |
| 3 | Male | 45 | Hyperglycemia | Right External Capsule Hemorrhage | 3 | 18 | 9 |
| 4 | Male | 60 | Atrial Fibrillation | Right Fronto-Parietal Junction and Temporal Lobe Hemorrhage | 3 | 8 | 24 |
| 5 | Female | 56 | Hyperlipidemia | Right Temporal Lobe, Basal Ganglia, Near Lateral Ventricle Hemorrhage | 6 | 5 | 38 |
| 6 | Male | 53 | Small Artery Sclerosis | Right Basal Ganglia Hemorrhage | 4 | 3 | 24 |
| 7 | Male | 69 | Hypertension | Right Basal Ganglia Hemorrhage | 5 | 6 | 5 |
| 8 | Female | 37 | Liver Dysfunction | Right Basal Ganglia Hemorrhage | 5 | 4 | 18 |
| 9 | Male | 75 | Hypertension | Right Basal Ganglia Hemorrhage | 4 | 9 | 16 |
| 10 | Female | 72 | Fatty Liver, Hypertension | Corpus Callosum, Left Lateral Ventricle | 5 | 10 | 29 |
| 11 | Male | 52 | Thyroid Nodule | Right Lateral Ventricle | 3 | 12 | 15 |
| 12 | Male | 66 | Hyperlipidemia, Hypertension | Right Basal Ganglia | 5 | 6 | 7 |
| 13 | Female | 75 | Left Internal Carotid Artery Occlusion | Left Basal Ganglia | 5 | 5 | 6 |
| 14 | Female | 46 | Hypertension | Right Basal Ganglia, Right Temporo-Frontal Lobe | 6 | 12 | 21 |
| 15 | Male | 72 | Cerebrovascular Stenosis | Parietal Lobe | 4 | 16 | 7 |
| 16 | Male | 66 | Hypertension, Atherosclerosis | Right External Capsule Hemorrhage | 3 | 21 | 6 |
| 17 | Female | 64 | Hypertension | Right Fronto-Parietal Junction and Temporal Lobe Hemorrhage | 6 | 18 | 33 |
| 18 | Female | 58 | Left Internal Carotid Artery Occlusion | Right External Capsule Hemorrhage | 4 | 14 | 25 |
| 19 | Male | 66 | Atherosclerosis | Right Basal Ganglia Hemorrhage | 3 | 21 | 6 |
| 20 | Female | 75 | Atherosclerosis | Right Basal Ganglia Hemorrhage | 6 | 16 | 27 |
| 21 | Female | 57 | Small Artery Sclerosis | Right Basal Ganglia Hemorrhage | 6 | 18 | 33 |
| 22 | Male | 57 | Hypertension | Right Fronto-Parietal Junction and Temporal Lobe Hemorrhage | 4 | 18 | 3 |
| 23 | Male | 75 | Hypertension | Right Basal Ganglia Hemorrhage | 6 | 15 | 4 |
| 24 | Female | 67 | Thyroid Nodule | Right Basal Ganglia Hemorrhage | 3 | 21 | 12 |
| 25 | Male | 55 | Atrial Fibrillation | Right Basal Ganglia Hemorrhage | 3 | 10 | 4 |
| 26 | Male | 65 | Hyperlipidemia | Left Basal Ganglia | 5 | 14 | 3 |
| 27 | Male | 55 | Cerebrovascular Stenosis | Right Basal Ganglia Hemorrhage | 6 | 13 | 6 |
| 28 | Female | 55 | Atrial Fibrillation | Right External Capsule Hemorrhage | 6 | 17 | 10 |
| 29 | Male | 62 | Hypertension | Right Fronto-Parietal Junction and Temporal Lobe Hemorrhage | 4 | 13 | 9 |
| 30 | Male | 40 | Hypertension | Right Basal Ganglia Hemorrhage | 6 | 19 | 14 |
| 31 | Female | 42 | Hyperglycemia | Left Basal Ganglia | 6 | 18 | 23 |
| 32 | Female | 54 | Atrial Fibrillation | Right Temporal Lobe, Basal Ganglia, Near Lateral Ventricle Hemorrhage | 6 | 21 | 24 |
| 33 | Male | 49 | Small Artery Sclerosis | Right Temporal Lobe, Basal Ganglia, Near Lateral Ventricle Hemorrhage | 5 | 16 | 8 |
| 34 | Female | 63 | Thyroid Nodule | Right Lateral Ventricle | 5 | 15 | 11 |
| 35 | Female | 50 | Hypertension | Left Parieto-Occipital Lobe | 5 | 19 | 7 |
| 36 | Female | 45 | Liver Dysfunction | Right Fronto-Parietal Junction and Temporal Lobe Hemorrhage | 4 | 19 | 9 |
| 37 | Male | 74 | Hypertension | Right Fronto-Parietal Junction and Temporal Lobe Hemorrhage | 6 | 14 | 8 |
| 38 | Male | 57 | Liver Dysfunction | Corpus Callosum, Left Lateral Ventricle | 6 | 16 | 7 |
| 39 | Male | 60 | Cerebrovascular Stenosis | Right Basal Ganglia Hemorrhage | 3 | 18 | 13 |
| 40 | Male | 45 | Atrial Fibrillation | Left Basal Ganglia | 3 | 15 | 18 |
| 41 | Female | 54 | Hypertension | Right External Capsule Hemorrhage | 4 | 21 | 15 |
| 42 | Male | 61 | Atrial Fibrillation | Right Basal Ganglia Hemorrhage | 4 | 27 | 14 |
| 43 | Female | 48 | Cerebrovascular Stenosis | Corpus Callosum, Left Lateral Ventricle | 6 | 19 | 25 |
| 44 | Male | 63 | Hypertension | Right Lateral Ventricle | 3 | 16 | 31 |
| 45 | Female | 53 | Hypertension | Right Fronto-Parietal Junction and Temporal Lobe Hemorrhage | 3 | 24 | 17 |
| 46 | Male | 57 | Hypertension | Corpus Callosum, Left Lateral Ventricle | 4 | 23 | 22 |
| 47 | Female | 53 | Atrial Fibrillation | Left Basal Ganglia | 3 | 23 | 15 |
| 48 | Female | 78 | Fatty Liver | Right Basal Ganglia Hemorrhage | 4 | 24 | 8 |
| 49 | Male | 59 | Hypertension | Left Basal Ganglia | 5 | 20 | 5 |
| 50 | Male | 49 | Hypertension | Right Basal Ganglia Hemorrhage | 5 | 12 | 29 |
| 51 | Male | 68 | Hypertension | Right Basal Ganglia Hemorrhage | 3 | 15 | 16 |
| 52 | Male | 80 | Fatty Liver | / | / | / | / |
| 53 | Male | 71 | Atrial Fibrillation | / | / | / | / |
| 54 | Male | 69 | Atrial Fibrillation | / | / | / | / |
| 55 | Female | 67 | Hypertension | / | / | / | / |
| 56 | Female | 56 | Hypertension | / | / | / | / |
| 57 | Female | 79 | Type 2 Diabetes Mellitus | / | / | / | / |
| 58 | Female | 75 | Hyperlipidemia | / | / | / | / |
| 59 | Female | 57 | Hypertension, Type 2 Diabetes Mellitus | / | / | / | / |
| 60 | Male | 66 | Hypertension, Type 2 Diabetes Mellitus | / | / | / | / |
| 61 | Male | 42 | Hypertension | / | / | / | / |
| 62 | Female | 65 | Hypertension | / | / | / | / |
| 63 | Male | 61 | Small Artery Sclerosis | / | / | / | / |
| 64 | Male | 68 | Thyroid Nodule | / | / | / | / |
| 65 | Male | 42 | Lung Nodule | / | / | / | / |
| 66 | Male | 46 | Kidney Stone | / | / | / | / |
| 67 | Male | 39 | Liver Cyst | / | / | / | / |
| 68 | Male | 55 | Diabetes | / | / | / | / |
| 69 | Female | 52 | Diabetes | / | / | / | / |
| 70 | Female | 52 | Hypertension | / | / | / | / |
| 71 | Female | 38 | None | / | / | / | / |
| 72 | Male | 53 | Hyperlipidemia | / | / | / | / |
| 73 | Male | 65 | Coronary Artery Disease | / | / | / | / |
| 74 | Male | 80 | Coronary Artery Disease | / | / | / | / |
| 75 | Female | 63 | Diabetes | / | / | / | / |
| 76 | Female | 61 | Coronary Artery Disease | / | / | / | / |
| 77 | Female | 54 | Hypertension | / | / | / | / |
| 78 | Male | 53 | Hypertension | / | / | / | / |
| 79 | Female | 69 | Hypertension | / | / | / | / |
| 80 | Female | 62 | Hypertension | / | / | / | / |
| 81 | Male | 53 | Hypertension | / | / | / | / |
| 82 | Male | 49 | Hypertension | / | / | / | / |
| 83 | Male | 58 | Hyperlipidemia | / | / | / | / |
| 84 | Male | 54 | Hypertension | / | / | / | / |
| 85 | Male | 59 | Hypertension | / | / | / | / |
| 86 | Male | 57 | Diabetes | / | / | / | / |
| 87 | Male | 63 | Hypertension | / | / | / | / |
| 88 | Female | 47 | Hyperlipidemia | / | / | / | / |

**Table S2. Primer sequence**

| Genes | Primer sequence (5'-3') |
| --- | --- |
| *Actb*-F | 5'-GAGGGAAATCGTGCGTGAC-3' |
| *Actb*-R | 5'-GCATCGGAACCGCTCATT-3' |
| *Cirbp*-F | 5'- GGACTCAGCTTCGACACCAAC -3' |
| *Cirbp* -R | 5'- ATGGCGTCCTTAGCGTCATC -3' |
| *Mcr1*-F | 5'-CAAGGAAGGTTGGCATTTGT-3' |
| *Mcr1*-R | 5'-CCTTTCAGTCCTTTGCAAGC-3' |
| *Arg1*-F | 5'-TCACCTGAGCTTTGATGTCG-3' |
| *Arg1*-R | 5'-CTGAAAGGAGCCCTGTCTTG-3' |
| *Nos2*-F | 5'-CAAGCACCTTGGAAGAGGAG-3' |
| *Nos2*-R | 5'- AAGGCCAAACACAGCATACC -3' |
| *Tnf* -F | 5'- GTTCTATGGCCCAGACCCTCAC-3' |
| *Tnf* -R | 5'- GGCACCACTAGTTGGTTGTCTTTG-3' |
| *Il6a* -F | 5'- ATCCTCGCCTATACCCCTGC -3' |
| *Il6a* -R | 5'- GGATACGGTGGGGGAGAAGT -3' |
| *Il6* -F | 5'- TGTATGAACAGCGATGATG -3' |
| *Il6*-R | 5'- AGAAGACCAGAGCAGATT -3' |
| *Rela*-F | 5'-AGGCTTCTGGGCCTTATGTG-3' |
| *Rela*-R | 5'-TGCTTCTCTCGCCAGGAATAC-3' |
